# Astrocytic gatekeeping of neural circuitry and synaptic balance in an autism mouse model: mechanistic insights beyond *Gryllus bimaculatus* extract-derived therapy

**DOI:** 10.3389/fcell.2025.1677851

**Published:** 2025-11-24

**Authors:** Haesung Lee, Ngoc Buu Tran, Sook-Jeong Lee

**Affiliations:** Department of Bioactive Material Sciences and Research Center of Bioactive Materials, Jeonbuk National University, Jeonju, Republic of Korea

**Keywords:** astrocyte dysfunction, autism spectrum disorder, excitation/inhibition balance, glial-neuronal interaction, natural extract, synaptic regulation

## Abstract

**Background:**

Autism spectrum disorder (ASD) is characterized by impaired synaptic development and disrupted excitation/inhibition (E/I) balance. While neuronal mechanisms have been extensively studied, accumulating evidence indicates that glial cells—particularly astrocytes—play a crucial role in maintaining synaptic homeostasis and regulating neurotransmission during brain development. However, the functional contribution of astrocytes to ASD pathogenesis remains insufficiently defined.

**Methods:**

This study aimed to explore astrocyte-mediated regulation of E/I balance in ASD using *Gryllus bimaculatus* (Gb) extract as a neuroprotective biological probe. A valproic acid (VPA)-induced ASD mouse model was established, and glial-neuronal interactions were assessed through analyses of neural progenitor cells, primary cortical neurons, astrocytes, and neuron-astrocyte co-cultures.

**Results:**

Gb extract reversed VPA-induced alterations in neural progenitor proliferation and differentiation and restored expression of key synaptic proteins (neuroligins, neurexin, synaptophysin). Notably, astrocyte-specific markers (GFAP, EAAT1/2) and E/I-related transporters (vGluT1, VGAT, GABA R1α, NMDA R1) were dysregulated in the ASD model and normalized by Gb treatment. Co-culture experiments demonstrated that astrocytes from VPA-treated animals, rather than neurons alone, were primarily responsible for E/I imbalance and synaptic abnormalities. Gb extract acted as a modulator of astrocytic function, restoring synaptic integrity and neurodevelopmental stability.

**Conclusion:**

Our findings underscore the pivotal role of astrocytes in the development and modulation of ASD-related synaptic pathology. Gb extract served as a valuable biological tool to reveal glial contributions to synaptic regulation and E/I balance. These insights support targeting astrocytic pathways with Gb extract as a novel therapeutic strategy for ASD.

## Introduction

Autism spectrum disorder (ASD) is a complex neurodevelopmental disorder characterized by deficits in social communication, repetitive behaviors, restricted interests, and challenges in social engagement (Lord et al., 2018; Morrison-Levy et al., 2018; Bi et al., 2018). Its prevalence has steadily increased over the past decades, creating a significant public health concern (Lord et al., 2018). Early postnatal identification and diagnosis of ASD remain difficult due to the heterogeneity of symptoms and their developmental emergence (Zablotsky et al., 2015).

Genetic factors are known to play a critical role in ASD etiology, with mutations in genes encoding synaptic proteins, such as neurexins (NRXNs), neuroligins (NLGNs), postsynaptic density protein 95 (PSD95), and SHANK family proteins (Talebizadeh et al., 2004; Yan et al., 2005; Guang et al., 2018; Gilbert and Man, 2017). Disruptions in these proteins impair synapse formation, stability, and plasticity, leading to excitatory/inhibitory (E/I) imbalances that are critical for the differentiation of glutamatergic and GABAergic neurons (Kim et al., 2014; Lenart et al., 2020; Kumamaru et al., 2014; Horder et al., 2018).

Environmental factors, particulary during pregnancy, further influence ASD risk. Maternal depression, exposure to teratogens, and other prenatal insults can negatively impact fetal brain development, resulting in delayed neurogenesis or structural anomalies (chaste and Leboyer, 2012; Deckmann et al., 2018; Chateauvieux et al., 2010; Romoli et al., 2019; Qiu et al., 2020). Among environmental factors, prenatal exposure to valproic acid (VPA) is widely utilized as a chemical model of ASD due to its ability to alter neuronal progenitor cell (NPC) proliferation, delay neurogenesis, and increase neuronal density in the fetal brain (Go et al., 2011; Go et al., 2012; Eissa et al., 2018). These models provide robust platforms to evaluate both the pathophysiology of ASD and potential therapeutic interventions (Abhishek et al., 2022).

Glial cells, particularly astrocytes, play an essential role in maintaining neural circuit integrity, regulating synaptic function, buffering extracellular ions, and protecting neurons from excitotoxicity (Verkhratsky and Nedergaard, 2018). Alterations in glial activity and morphology, including changes in glial fibrillary acidic protein (GFAP) expression, have been documented in individuals with ASD and in preclinical models (Edmonson et al., 2014; Gupta et al., 2014). Dysregulated glial function contributes to neuroinflammation, E/I imbalance, and impaired synaptic plasticity, highlightling the critical role of neuron-glia interactions in ASD pathogenesis (Gzielo et al., 2019).

Despite advances in understanding ASD biology, current pharmacological strategeis are largely limited to alleviating secondary symptoms, such as irritability, aggression, or anxiety. FDA-approved drugs, including risperidone and aripiprazole, improve behavioral disturbances but do not address core deficits or alter disease progression, and their long-term safety remains a concern (McPheeters et al., 2011; Owen et al., 2009). Clinical trials are further complicated by patient heterogeneity, comorbidities, and a lack of reliable biomarkers for assessing treatment efficacy (Masi et al., 2017). These limitations underscore the urgent need for innovative, disease-modifying therapeis that target the underlying cellular and molecular dysfunctions in ASD. In this context, natural bioactive compounds have emerged as promising therapeutic candidates due to their multi-target mechanisms, which include antioxidant, anti-inflammatory, neuroprotective, and neuromodulatory activities (Bhandari and Kuhad, 2015; Russo, 2009; Cruz-Martins et al., 2021). Compounds such as polyphenols, flavonoids, omega-3 fatty acids, and carotenoids can reduce oxidative stress via Nrf 2 signaling, suppress neuroinflammation through NF-kB pathways, enhance synaptic plasticity by upregulating brain-derived neurotrophic factor (BDNF), and influence the microbiota-gut-brain axis (Hsiao et al., 2013). These characteristics provide a compelling rationale for exploring natural compounds as interventions capable of modifying disease progression rather than merely managing symptoms.

Among natural sources, insect-derived compounds offer a novel and underexplored avenue for neuroprotective therapy. Insects are nutrient-rich, sustainable, and a rich reservoir of bioactive molecules with anti-inflammatory, antioxidant, and neuromodulatory effects (Rumpold and Schluter, 2013; Ahn et al., 2014; Hwang et al., 2019; Park et al., 2020). Emerging evidence indicates that insect extracts can protect nervous system integrity, regulate neuron-glia interactions, and preserve blood-brain barrier function in conditions such as epilepsy and aging (Tran and Lee, 2023b; Buu Tran et al., 2024). This positions insect-derived compounds as innovative therapeutic candidates capable of addressing multiple pathogenic mechanisms in ASD, from oxidative stress to synaptic dysregulation.

In particular, preclinical studies using VPA-induced ASD mouse models and *in vitro* systems provide evidence that *Gryllus bimaculatus* extract can regulate neuronal plasticity and stabilize neural circuits. By directly targeting neuronal plasticity, Gb extract represents a translationally relevant therapeutic strategy with potential disease-modifying effects beyond conventional symptom-focused interventions.

This study, therefore, investigates the effects of an aqueous extract of Gb on astrocytic regulation of neural circuitry and synaptic balance in an ASD mouse model. By integrating neuroprotective and synapse-stabilizing properties, this research aims to elucidate mechanistic insights that extend beyond the extract itself, highlighting the broader potential of insect-derived bioactive compounds in ASD therapeutics. These finidings may pave the way for novel intervention strategies that not only address core pathophysiolocial features but also enhance translational applicability for future clinical development.

## Methods

### Chemicals and antibodies

The sodium salt of valproic acid (VPA; #P4543) was obtained from Sigma-Aldrich (St. Louis, MO, USA), and Gb powder was supplied by Purnae Company (Sejong, Korea). Antibodies against synaptic markers, including NLGN1 (#NBP2-42192), NLGN2 (#NBP2-41299), NLGN3 (#NBP2-42200), SHANK3 (#NBP1-47610), the NMDA receptor NR1 subunit (#NB300-114) from Novus Biologicals (Centennial, CO, United States) and NRXN1 (#PA5-79764) was from Thermo Fisher Scientific (Waltham, MA, United States). Vesicular glutamate transporter 1 (vGluT1; #GTX133148), vesicular GABA transporter (VGAT; #GTX101908), synaptophysin (#GTX100865), anti-GABA A receptor alpha 1 (#ab252430), metabotropic glutamate receptor subunits GRM2/GRM3 (#CSB-PA009022) and GRM5 (#CSB-PA003236) were obtained from Cusabio Technology (Huston, TX, United States). Further, antibodies against glial markers and neuron markers such as GFAP (GA5; #3670T) from Cell Signaling Technology (Danvers, MA, USA), nestin (#14-5843-82) from Thermo Fisher Scientific (Waltham, MA, USA), ionized calcium-binding adaptor molecule 1 (Iba1; #GTX100042), and oligodendrocyte transcription factor 2 (OLIG2; #GTX132732) from GeneTex Inc. (Irvine, CA, United States) and anti-beta III tubulin (#ab78078) from Abcam (Cambridge, UK) were used. Proliferation marker Ki-67 (SP6; #MA5-14520) was purchased from Thermo Fisher Scientific. Housekeeping proteins were probed using antibodies against β-actin (#4967S; Cell Signaling Technology) and α-tubulin (#BS1699; Bioworld Technology, Nanjing, China).

### Establishment of a VPA-induced autism spectrum disorder model in mice

**FIGURE 1:**
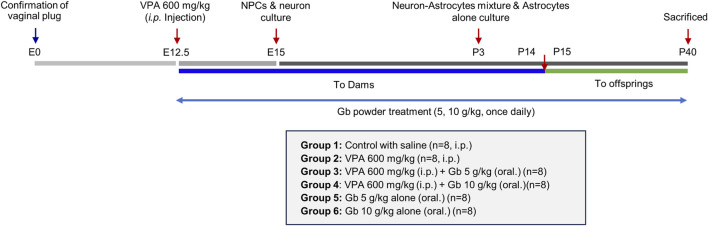
Schematic overview of experimental timeline from fetal development to the early postnatal period in mice. This diagram illustrates the sequence of procesures used to establish the valproic acid (VPA)-induced autism model, the administration schedule of *Gryllus bimaculatus* (Gb) extract, and the designated sampling points for subsequent analyses. Detection of a vaginal plug was defined as embryonic day 0 (E0). VPA (600 mg/kg) was administrated intraperitoneally on E12.5, and Gb extract treatment commenced on the same day. Dams received Gb extract from E12.5 through postnatal day 14 (P14). Following weaning, Gb extract was administrated directly to the offsprings from P15 to P40. Neural progenitor cell (NPC) and neuron cultures were established on E15, whereas astrocytes cultures were generated from pups on P3. On P40, the final day of the experimental period, offspring were euthanized and brain tissues were collected for *in vitro* and biochemical analyses.

Eight-week-old male and female ICR mice (20–25 g) were obtained from Samtaco (Suwon, South Korea). The animals had *ad libitum* access to water and a standard rodent chow diet. They were housed under controlled conditions at a temperature of 24 ± 2 °C, humidity of 50% ± 10%, and a 12-h light/dark cycle. All efforts were made to minimize the number of animals used and to reduce stress and suffering. The mice underwent a 1-week acclimation period before the experiment commenced. Subsequently, male and female mice were paired for mating, with the presence of a vaginal plug marking embryonic day 0 (E0). Pregnant mice were maintained for 12 days before being assigned to one of the six experimental groups: (1) control group (n = 8), (2) VPA 600 mg/kg (n = 8), (3) VPA 600 mg/kg + 5 g/kg Gb extract (n = 8), (4) VPA 600 mg/kg + 10 g/kg Gb extract (n = 8), (5) 5 g/kg Gb extract (n = 8), and (6) 10 g/kg Gb extract (n = 8). On E12.5, pregnant females received an Intraperitoneal injection of either 600 mg/kg VPA or normal saline (0.9%). Following administration, only the pregnant mice not designated for embryo collection were returned to their cages and left undisturbed until natural parturition, which typically occurred between E21 and E23, as illustrated in Figure 1, whereas those assigned for embryo collection were humanely euthanized at the designated embryonic stages. The offspring in each group were nursed by their dams for 2 weeks until P14. After weaning, when they were able to intake a solid food freely, the Gb extract was orally administered to the offspring using the same procedure as that applied to the dams. Subsequently, the dams were sacrificed. Gb extracts were administered with or without concurrent VPA treatment from E12.5 until P40, as illustrated in Figure 1. All procedures involving animals were reviewed and approved by the Animal Experimentation Ethics Committee of Jeonbuk National University [JBNU 2024-071].

### Drug treatments

The amount of Gb extract used in this study was determined based on previously published results (Tran et al., 2023a; Yu et al., 2020). Pregnant ICR mice were randomly assigned to six experimental groups: (1) control group (n = 8), (2) VPA 600 mg/kg (n = 8), (3) VPA 600 mg/kg + 5 g/kg Gb extract (n = 8), (4) VPA 600 mg/kg + 10 g/kg Gb extract (n = 8), (5) 5 g/kg Gb extract (n = 8), and (6) 10 g/kg Gb extract (n = 8). On embryonic day 12.5 (E12.5), each dam received an intraperitoneal injection of either 600 mg/kg valproic acid (VPA) or normal saline (0.9%). Following administration, pregnant mice were either humanely euthanized at the designated embryonic stages for embryo collection or, if not assigned for this purpose, were returned to their cages and allowed to continue pregnancy undisturbed until natural parturition (E21–E23). Gb extract was prepared by extracting dry Gb powder in distilled water (1:10 w/v) at 60 °C for 12 h, followed by filtration (0.2 μm pore size), concentration under reduced pressure, and freeze-drying. Extracts were stored at 4 °C until use.

$$\begin{array}{ll} & \text{Yield }\left(\%\right)=\left(\text{Weight after drying}/\right.\\ & \;\left.\text{× Weight of powder used for extraction}\right)\times 100 \end{array}$$

Using this method, the extraction yield of the water-soluble Gb powder extract was determined to be 15.33%. Furthermore, compositional analyses of the extracts were conducted at the Center for University-wide Research Facilities (CURF), Jeonbuk National University, with the corresponding results documented in the supplementary data.

Male pups were weaned and separated by sex at postnatal day 21 (P21). From E12.5 to P40, mice in designated groups received a daily oral dose of Gb extract (5 or 10 g/kg, 0.1 mL). Controls received matched volumes of water. The experimental timeline is summarized in Figure 1.

### Cell culture protocols

Embryos and pups obtained from selected pregnant dams within each experimental group (n = 8 per group) were randomly pooled to establish primary cultures, thereby minimizing litter-specific variability while preserving representative biological diversity among the offspring.

### Neural progenitor cell culture

Neural progenitor cells (NPCs) were isolated from embryonic day 15 (E15) mouse brains using a modified protocol based on Hutton and Pevny (2008). For each experimental group, four embryos obtained from partially pooled dams were used for NPC culture, while the remaining embryos from the same dams were used to establish primary neuronal cultures. All pregnant dams used for embryo collection were humanely euthanized immediately after tissue harvest in accordance with approved institutional ethical guidelines. Briefly, whole brains were enzymatically dissociated with Accutase for 20 min. Cells from all four hemispheres were plated in T-75 flasks containing DMEM/F-12 medium supplemented with B-27, 20 ng/mL epidermal growth factor (EGF), and 10 ng/mL fibroblast growth factor (FGF) to support the growth of NPCs. Neurosphere formation was monitored daily. After 14 days, free-floating neurospheres were collected and centrifuged for passaging. Neurospheres at passage two were cultured for another 14 days to promote further proliferation. For differentiation, neurospheres were transferred to DMEM/F-12 medium with B-27% and 2% horse serum.

### Astrocyte culture

Astrocyte cultures were established from the neocortex of P3 mice following the protocol described by McCarthy and de Vellis (1980). For each experimental group, astrocyte cultures were established from six pups derived from randomly selected litters. All donor animals used for pup collection were humanely sacrificed immediately after tissue retrieval in compliance with institutional animal care and use guidelines. In brief, neonatal cortices were dissociated, and 0.5 hemisphere per well was seeded into poly-L-lysine-coated 24-well plates. The culture medium consisted of DMEM supplemented with 2 mM glutamine, 7% fetal bovine serum (FBS), and 7% HS. The cells were allowed to mature, and experiments were conducted 14 days after seeding. The purity of the astrocytes was determined by staining with a GFAP antibody, and DAPI was used as a nuclear counterstain. Nearly 100% of the cultured cells were GFAP-positive.

### Neuron culture

Pure neuronal cultures were derived from the cortices of E15 mice. Dissociated cortical cells were plated onto laminin-coated 24-well plates at a density of four hemispheres per plate using neurobasal medium supplemented with B-27 serum. To inhibit the proliferation of non-neuronal cells, 10 μM cytosine arabinoside (AraC) was added 5 days post-seeding.

### Mixed cortical cell culture

Mixed cultures containing both neurons and astrocytes were prepared using fetal mice at E15 and neonatal mice at P3, as described by Choi et al. (1987). For the establishment of mixed cortical cell cultures, neonatal mice at P3 were used initially. Cortical cells were isolated from these P3 mice brain and seeded onto poly-L-lysine-coated 24-well plates, where they were cultured for approximately 2 weeks until reaching confluency. Upon confirmation of astrocyte confluency, cortical cells isolated from E15 mice were subsequently seeded directly onto the astrocyte monolayer containing DMEM supplemented with 5% FBS, 5% HS, and 2 mM glutamine. To prevent the proliferation of non-neuronal cells, AraC was added 5 days post-seeding.

### Immunocytochemistry and image acquisition analysis

Cultured cells were fixed with 4% paraformaldehyde for 30 min, permeabilized with 0.1% Triton X-100 for 15 min, and blocked with 1% bovine serum albumin (BSA) for 1 h at room temperature. Primary antibodies were incubated overnight at 4 °C, followed by a 1-h incubation with secondary antibodies at room temperature. Cell nuclei were counterstained with 4′,6-diamidino-2-phenylindole (DAPI).

Confocal imaging was performed using a Carl Zeiss confocal microscope (LSM 880 with Airyscan; Oberkochen, Germany). Laser excitation wavelengths and emission filters were optimized for Alexa Fluor 488, Alexa Fluor 555, Alexa Fluor 647, and DAPI. Z-stack images were captured with a step size of 0.3 μm to enable three-dimensional visualization of cellular structures.

For synaptic immunostaining, neuronal cells were co-labeled with anti-synaptophysin and anti-Tuj-1 antibodies. Synaptic puncta and dendritic trees were determined by subtracting the total Tuj-1-labeled area. Quantitative analysis of protein-positive signals was conducted using ImageJ software (NIH). Briefly, all images were converted to 8-bit grayscale, and positive immunoreactive regions were identified by applying a fixed gray threshold, which was empirically determined based on control images to optimally distinguish signal from background. This threshold was then consistently applied across all experimental groups. Additionally, brightness and contrast adjustments were uniformly applied to all images to ensure comparability between conditions.

### Western blot analysis

Protein extracts prepared from both cultured cells and brain homogenates originating from the same randomly combined samples were utilized to maintain procedural uniformity across all analyses. In brief, the samples were lysed in RIPA buffer composed of 150 mM NaCl, 1% Triton X-100, 0.5% sodium deoxycholate, 0.1% SDS, and 50 mM Tris-HCl (pH 8.0), followed by quantification of total protein using the bicinchoninic acid (BCA) assay (Thermo Fisher Scientific). Equal amounts of protein were separated by SDS-polyacrylamide gel electrophoresis and transferred to polyvinylidene difluoride membranes. Membranes were blocked for 1 h at room temperature with Tris-buffered saline containing 0.1% and 5% skim milk, then incubated overnight at 4 °C with primary antibodies (1:1000) in phosphate-buffered saline with Tween 20 (PBS-T) containing 1% BSA. Following primary antibody incubation, membranes were incubated for 1 h at room temperature with horseradish peroxidase-conjugated secondary antibodies (1:10,000) in PBS-T containing 1% BSA. Immunoreactive proteins were visualized using an enhanced kit (Thermo Scientific™ West Femto maximum sensitivity substrate, #34095) and imaged using the iBright CL1000 imaging system (Thermo Fisher Scientific). Protein band intensity was quantified via densitometric analysis using ImageJ software, with normalization to the corresponding α-tubulin or total protein bands.

### Statistical analysis

All statistical analyses were performed using GraphPad Prism 7 software (San Diego, CA, United States). Data are presented as the mean ± standard error of the mean (SEM). For comparisons between two groups, Student’s *t*-tests were employed. For experiments involving six groups with multiple comparisons, one-way analysis of variance (ANOVA) was conducted, followed by the Tukey’s multiple comparison test. Statistical significance is indicated as follows: *p < 0.05, **p < 0.01, ***p < 0.001 compared with control; #*p* < 0.05, ##*p* < 0.01, ###*p* < 0.001 compared with VPA alone; *NS*, not significant. Unless otherwise specified in the figure legends, non-significant results are reported as *NS*.

## Results

### Gb extracts restore VPA-induced abnormalities in neural progenitor cell proliferation and differentiation in fetal mice

**FIGURE 2:**
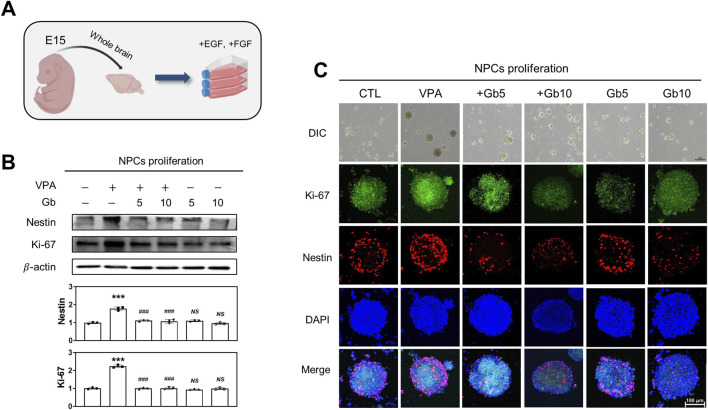
Effect of *Gryllus bimaculatus* (Gb) extract on the proliferation of neuronal progenitor cells (NPCs) isolated from embryonic mice exposed to valproic acid (VPA). **(A)** Schematic representation of NPC cultures derived from embryonic mouse brains, illustrating the induction of cellular proliferation. **(B)** Western blot analysis of NPC lysates for Nestin and Ki-67. Equal amounts of protein were loaded into each lane, with β-actin serving as a loading control. The bars represent fold-changes in the densitometric values of Nestin and Ki-67 bands relative to the corresponding β-actin band densities. Control values were normalized to 1 (mean ± SEM, n = 3; ***p < 0.001 compared with control; ^###^ *p* < 0.001 compared with VPA alone; *ns*, not significant). **(C)** Differential interference contrast (DIC) and confocal fluorescence microscopy images of Ki-67 and Nestin. NPCs were passaged and cultured over 14 days to facilitate neurosphere formation. Scale bar: 200 μm. After fixation, neurosheres were immunostained for Ki-67 (green) and Nestin (red), with nuclei counterstained using DAPI (blue). Scale bar: 100 μm. Experimental groups included CTL (saline, n = 8); VPA (600 mg/kg VPA, n = 8); VPA + Gb*5* (600 mg/kg VPA + 5 g/kg Gb extract, n = 8); VPA + Gb*10* (600 mg/kg VPA + 10 g/kg Gb extract, n = 8); Gb*5* (5 g/kg Gb extract, n = 8); Gb*10* (10 g/kg Gb extract, n = 8).

**FIGURE 3:**
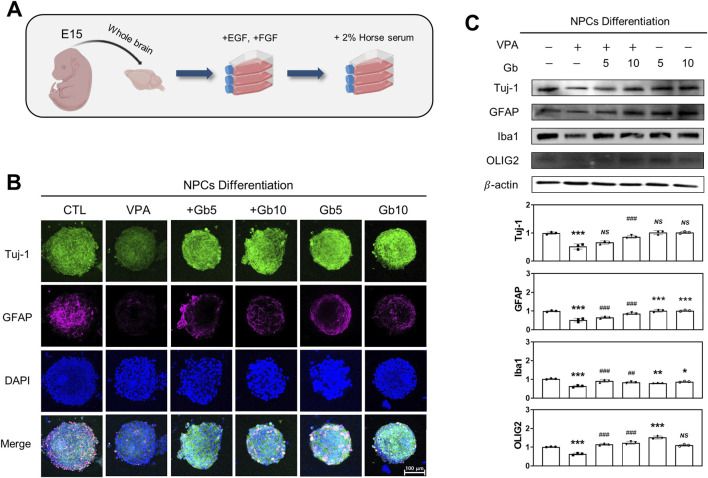
Influence of *Gryllus bimaculatus* (Gb) extracts on the differentiation of neuronal progenitor cells (NPCs) derived from embryonic mice treated with valproic acid (VPA). **(A)** Schematic representation of the differentiation process of NPCs from embryonic mouse brains. **(B)** Confocal fluorescence microscopy images of differentiated NPCs. After neurosphere formation was confirmed on *in vitro* day 15, the medium was replaced with 1% horse serum-supplemented medium to promote differentiation. Five days post-differentiation, the cells were fixed and subjected to immunofluorescence staining. Cells were stained for Tuj-1 (green) and GFAP (purple), with nuclei counterstained using DAPI (blue). Scale bar: 100 μm. **(C)** Western blot analysis of Tuj-1, GFAP, Iba1, and OLIG2 in differentiated NPC lysates. Equal amounts of protein were loaded per lane, with β-actin used as a loading control. The bars represent fold-changes in the densitometric values of Tuj-1, GFAP, Iba1, and OLIG2 bands relative to β-actin. Control values were normalized to 1 (mean ± SEM, n = 3; *p < 0.05, **p < 0.01, ***p < 0.001 compared with control; ^##^ *p* < 0.01, ^###^ *p* < 0.001 compared with VPA alone; *ns*, not significant). Experimental groups included CTL (saline, n = 8); VPA (600 mg/kg VPA, n = 8); VPA + Gb*5* (600 mg/kg VPA + 5 g/kg Gb extract, n = 8); VPA + Gb*10* (600 mg/kg VPA + 10 g/kg Gb extract, n = 8); Gb*5* (5 g/kg Gb extract, n = 8); Gb*10* (10 g/kg Gb extract, n = 8).

To assess the impact of Gb extract on embryonic brain development in fetuses exposed to VPA, NPCs were isolated from E15 embryos of pregnant mice and evaluated for proliferation and differentiation, as schematically illustrated in Figures 2A, 3A. Differences in NPC proliferation were analyzed by measuring the expression levels of nestin and Ki-67, well-established markers of NPC proliferation and neural development, respectively. Western blotting and immunocytochemistry revealed a significant increase in nestin and Ki-67 expression levels in the VPA treatment group compared with the control and Gb extract-only groups (Figures 2B,C). Notably, VPA 600 mg/kg + Gb extract 5 g/kg group and VPA 600 mg/kg + Gb extract 10 g/kg group was fully restored the expression of these markers to levels comparable to those of the control (*p* ≤ 0.001, Figures 2B,C). Furthermore, the increased nestin and Ki-67 expression observed in the VPA group, indicative of enhanced cellular proliferation, was normalized to control levels following Gb extract treatment in 5 g/kg group and 10 g/kg group (*p* ≤ 0.001, Figures 2B,C).

In addition to NPC proliferation, the effects of Gb extract on VPA-induced NPC differentiation were also examined. After a 14-day proliferation period, NPC differentiation was induced by supplementing the culture medium with 2% HS (Figure 3A). Differentiated NPCs were immunostained for Tuj-1, GFAP, Iba1, and Olig2, which serves as markers for mature neurons, astrocytes, microglia, and oligodendrocytes, respectively. Quantitative and qualitative analyses using western blotting and immunocytochemistry revealed significantly reduced expression of Tuj-1, GFAP, Iba1, and OLIG2 in NPCs derived from VPA-treated embryos compared with both the control and Gb extract-treated groups (*p* ≤ 0.001, Figures 3B,C). Importantly, Gb extract treatment effectively mitigated the VPA-induced inhibition of NPC differentiation, restoring the expression of these markers to control levels (Tuj-1, *NS*; GFAP, *p* ≤ 0.001; Iba1, *p* ≤ 0.01; OLIG2, *p* ≤ 0.001, Figures 3B,C).

### Gb extracts mitigate abnormal neuronal network and synaptic deficits in a VPA-induced ASD mouse model

**FIGURE 4:**
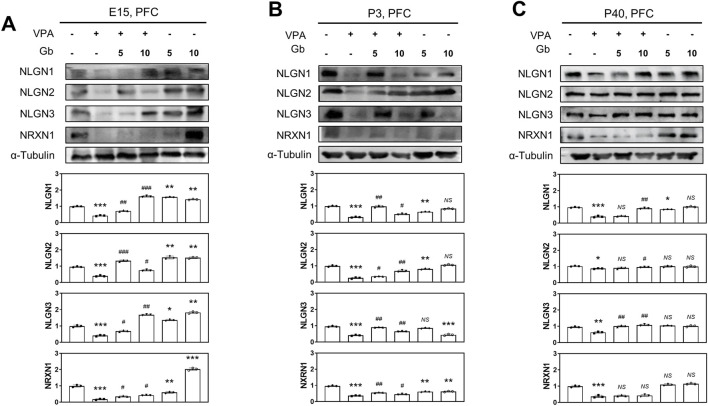
Neuroprotective effects of *Gryllus bimaculatus* (Gb) extract on synaptic development impairments in the valproic acid (VPA)-induced autism spectrum disorder (ASD) mouse model. Immunoblot analyses of NLGN1, NLGN2, NLGN3, and NRXN1 proteins were performed on prefrontal cortex (PFC) tissue lysates collected at E15 **(A)**, P3 **(B)**, and P40 **(C)** from mice subjected to various combination treatments. Experimental groups included CTL (saline, n = 8); VPA (600 mg/kg VPA, n = 8); VPA + Gb*5* (600 mg/kg VPA + 5 g/kg Gb extract, n = 8); VPA + Gb*10* (600 mg/kg VPA + 10 g/kg Gb extract, n = 8); Gb*5* (5 g/kg Gb extract, n = 8); Gb*10* (10 g/kg Gb extract, n = 8). Control values were normalized to 1 (mean ± SEM, n = 3; *p < 0.05, **p < 0.01, ***p < 0.001 compared with control; ^#^ *p* < 0.05, ^##^ *p* < 0.01, ^###^ *p* < 0.001 compared with VPA alone; *ns*, not significant).

NLGNs and NRXNs play a crucial role in the differentiation, maturation, and stabilization of both inhibitory and excitatory synapses. The findings revealed a significant reduction in the expression of NLGN family proteins (NLGN1, NLGN2, and NLGN3) and NRXN1 in the VPA-treated groups at E14, P3, and P40 (E14 and P3, *p* ≤ 0.001; P40, *p* ≤ 0.001, *p* ≤ 0.05, *p* ≤ 0.01 and *p* ≤ 0.001, respectively, Figures 4A–C). No significant changes were observed in NLGNs and NRXN1 expression in the Gb extract-only group compared with the control group. However, Gb extract treatments with VPA 600 mg/kg successfully restored the expression of NLGNs and NRXN1 at E14 (*p* ≤ 0.001, *p* ≤ 0.001, *p* ≤ 0.01 and *p* ≤ 0.05, respectively), P3 (*p* ≤ 0.01, *p* ≤ 0.01, *p* ≤ 0.01 and *p* ≤ 0.01, respectively), and P40 (*p* ≤ 0.01, *p* ≤ 0.05, *p* ≤ 0.01, *NS*, respectively) (Figures 4A–C).

**FIGURE 5:**
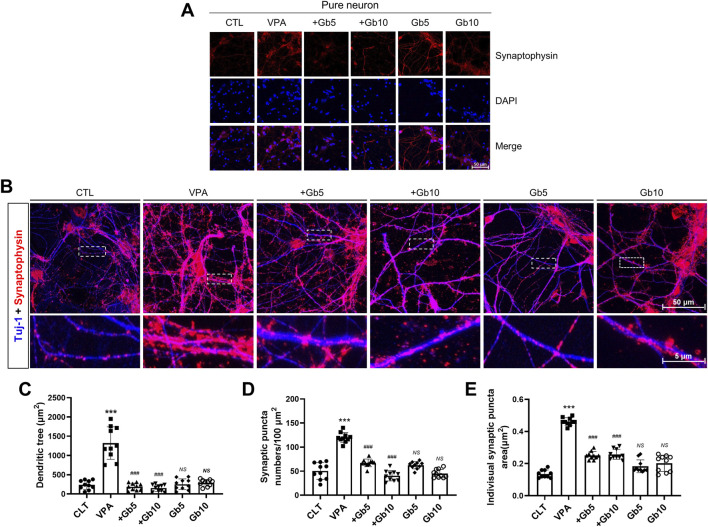
Protective effects of *Gryllus bimaculatus* (Gb) extract on synaptic activity and synaptogenesis in cortical neurons of mice exposed to valproic acid (VPA). **(A)** Confocal microscopy images of cortical neurons from the experimental groups. Cells cultured for 7 days were fixed and immunostained for synaptophysin (red), with nuclei counterstained using DAPI (blue). Scale bar: 50 μm. **(B)** Immunostaining of cortical neurons for synaptophysin (red) and Tuj-1 (blue) to visualize synaptic patterns. The lower row shows magnified views of the areas marked by white dots. Quantification of **(C)** Tuj-1-positive dendritic tree area, **(D)** synaptophysin puncta number, and **(E)** individual synaptophysin puncta area in cortical neurons cultured from the different experimental groups. Experimental groups included CTL (saline, n = 8); VPA (600 mg/kg VPA, n = 8); VPA + Gb*5* (600 mg/kg VPA + 5 g/kg Gb extract, n = 8); VPA + Gb*10* (600 mg/kg VPA + 10 g/kg Gb extract, n = 8); Gb*5* (5 g/kg Gb extract, n = 8); Gb*10* (10 g/kg Gb extract, n = 8). Control values were normalized to 1 (mean ± SEM, n = 3; ***p < 0.001 compared with control; ^###^ *p* < 0.001 compared with VPA alone; *ns*, not significant).

Synaptogenesis, a vital process throughout life, is particularly accelerated during early brain development. To explore synapse formation, autism was induced in mice through VPA treatment, and cortical neurons derived from E15 fetuses were analyzed. Immunofluorescence staining of cortical neurons using synaptophysin, a well-established marker for presynaptic vesicle membranes, revealed significantly elevated synaptophysin expression in the VPA group compared with both the control and Gb extract-treated groups (5 g/kg and 10 g/kg) (Figure 5A). Gb extract 5 g/kg group and 10 g/kg group nearly normalized VPA-induced synaptophysin abnormalities, demonstrating a protective effect at both doses (Figure 5A).

To further analyze patterns of neuronal and synaptic development, co-immunofluorescence staining for Tuj-1 and synaptophysin was performed. Quantitative analysis was conducted used to measure Tuj-1-positive signals (Figures 5B–E). The average dendritic area in the VPA group was 1322.90 μm^2^, approximately six times larger than the control group (239.62 μm^2^, *p* ≤ 0.001) (Figures 5B,C). In contrast, 5 g/kg or 10 g/kg of Gb extract resulted in average dendritic areas of 189.53 μm^2^ and 155.83 μm^2^, respectively, representing approximately 7-fold and 8-fold reductions compared with the VPA group (*p* ≤ 0.001, Figures 5B,C). The Gb extract-only groups (5 g/kg and 10 g/kg) did not show significant differences from the control group, with dendritic areas of 253.92 μm^2^ and 290.98 μm^2^, respectively (Figures 5B,C).

Synaptophysin-positive synaptic puncta were quantified within the defined ROIs. The VPA group exhibited an average of 120 puncta, more than twice the 50 puncta observed in the control group (*p* ≤ 0.001, Figures 5B,D). While the Gb extract-only groups did not exhibit significant differences from the control, Gb extract with VPA 600 mg/kg groups significantly reduced the synaptic puncta counts to 67 and 40 for the 5 g/kg and 10 g/kg treatments, respectively, compared with the VPA group (*p* ≤ 0.001, Figures 5B,D). To evaluate synaptogenesis in the VPA-treated group, the size of presynaptic vesicles was measured based on synaptophysin- and Tuj-1-positive neuron signals. The average vesicle size in the control group was 0.13 μm^2^, whereas it was 0.46 μm^2^ in the VPA group, an approximately threefold increase (*p* ≤ 0.001, Figure 5E). Gb extract with VPA 600 mg/kg groups at both concentrations significantly reduced vesicle size to 0.25 μm^2^ (*p* ≤ 0.001, Figure 5E). The Gb extract-only groups had vesicle sizes of 0.18 μm^2^ and 0.20 μm^2^ for 5 g/kg and 10 g/kg treatments, respectively, which were not significantly different from the control group (Figure 5E).

### Neuroprotective effect of Gb extract on VPA-triggered E/I imbalance via altered glutamatergic/GABAergic receptor plasticity

The balance between E/I neuronal signaling is crucial for normal brain function, and disruptions in this balance has been implicated in VPA-induced ASD. This study investigated

Whether Gb extract can ameliorate VPA-induced E/I imbalance and examined the impact of VPA on E/I balance at different stages of brain development. To assess these effects, protein expression levels associated with glutamatergic and GABAergic receptors, as well as vesicles, were analyzed in the cortical brains of mice at E14, P3, and P40. GRM5, vGluT1, and NMDA receptor 1 (NMDA R1) serve as markers of excitatory neuronal activity, whereas GABA receptor 1α (GABA R1α) and VGAT are indicative of inhibitory neuronal activity.

**FIGURE 6:**
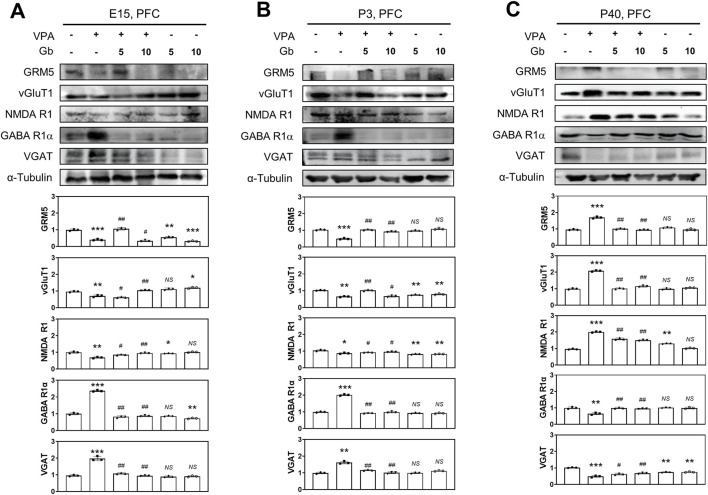
Protective effects of *Gryllus bimaculatus* (Gb) extract on abnormal expression levels of glutamatergic and GABAergic synaptic proteins in the valproic acid (VPA)-induced autism spectrum disorder (ASD) mouse brain tissues. Immunoblot analyses for GRM5, vGluT1, NMDA R1, GABA R1α, and VGAT proteins were performed on prefrontal cortex (PFC) tissue lysates collected at embryonic day 15 (E15) **(A)**, postnatal day 3 (P3) **(B)**, and P40 **(C)** from mice subjected to various treatment combinations. Experimental groups included CTL (saline, n = 8); VPA (600 mg/kg VPA, n = 8); VPA + Gb*5* (600 mg/kg VPA + 5 g/kg Gb extract, n = 8); VPA + Gb*10* (600 mg/kg VPA + 10 g/kg Gb extract, n = 8); Gb*5* (5 g/kg Gb extract, n = 8); Gb*10* (10 g/kg Gb extract, n = 8). Control values were normalized to 1 (mean ± SEM, n = 3; *p < 0.05, **p < 0.01, ***p < 0.001 compared with control; ^#^ *p* < 0.05, ^##^ *p* < 0.01, ^###^ *p* < 0.001 compared with VPA alone; *ns*, not significant).

Western blot analysis (Figure 6) revealed significantly reduced expression levels of NMDA R1, GRM5, and vGluT1 in the VPA treatment group, whereas GABA R1α and VGAT levels were markedly elevated in brain lysates at E14 (*p* ≤ 0.01, *p* ≤ 0.001, *p* ≤ 0.01, *p* ≤ 0.001 and *p* ≤ 0.001, respectively) and P3 (*p* ≤ 0.05, *p* ≤ 0.001, *p* ≤ 0.01, *p* ≤ 0.001 and *p* ≤ 0.01, respectively) (Figures 6A,B). In contrast, treatment with Gb extract alone did not significantly alter at the expression of NMDA R1, GRM5, vGluT1, GABA R1α, or VGAT compared with the control group in E14 (*p* ≤ 0.05, *p* ≤ 0.001, *p* ≤ 0.05, *p* ≤ 0.01 and *NS,* respectively) and P3 (*p* ≤ 0.01, *NS*, *p* ≤ 0.01, *NS* and *NS,* respectively) group (Figures 6A,B). Notably, by P40, expression patterns were reversed in the VPA-treated group, with NMDA R1, GRM5, and vGluT1 significantly elevated (*p* ≤ 0.001), whereas GABA R1α and VGAT were reduced (*p* ≤ 0.01 and *p* ≤ 0.001, respectively) (Figure 6C). 5 g/kg or 10 g/kg of Gb extract with VPA 600 mg/kg groups reduced the expression of NMDA R1 (E14, *p* ≤ 0.01; P3, *p* ≤ 0.05; P40, *p* ≤ 0.01), GRM5 (E14, *p* ≤ 0.001; P3, *p* ≤ 0.001; P40, *p* ≤ 0.001), and vGluT1 (E14, *p* ≤ 0.01; P3, *p* ≤ 0.01; P40, *p* ≤ 0.001), while normalizing GABA R1α (E14, *p* ≤ 0.01; P3, *p* ≤ 0.01; P40, *p* ≤ 0.01) and VGAT (E14, *p* ≤ 0.01; P3, *p* ≤ 0.01; P40, *p* ≤ 0.01) levels. No significant differences in these markers were observed in the Gb extract-only group compared with the control group (Figure 6C).

### A key role of astrocytes in E/I regulation: The protective role of Gb extract in restoring VPA-induced E/I imbalance

**FIGURE 7:**
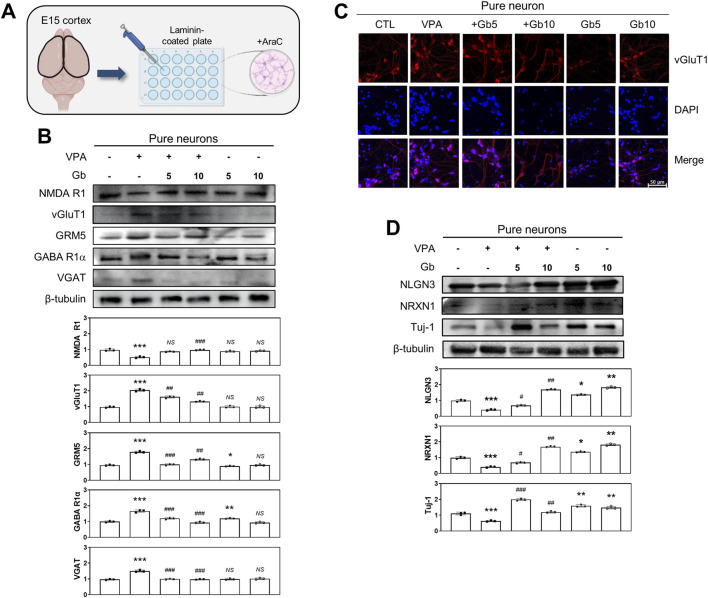
Regulatory effects of *Gryllus bimaculatus* (Gb) extract on excitatory and inhibitory neuronal activity in primary cortical neurons from valproic acid (VPA)-treated embryonic mice. **(A)** Schematic representation of primary cortical neuron cultures derived from embryonic mouse brains. Experimental groups included CTL (saline, n = 8); VPA (600 mg/kg VPA, n = 8); VPA + Gb*5* (600 mg/kg VPA + 5 g/kg Gb extract, n = 8); VPA + Gb*10* (600 mg/kg VPA + 10 g/kg Gb extract, n = 8); Gb*5* (5 g/kg Gb extract, n = 8); Gb*10* (10 g/kg Gb extract, n = 8). **(B,D)** Immunoblot analyses of NMDA R1, vGluT1, GRM5, GABA R1α, VGAT, NLGN3, NRXN1, and Tuj-1 in cultured primary cortical neuron lysates. Equal amounts of protein were loaded per lane, with β-tubulin used as a loading control. The bars represent fold-changes in the densitometric values of individual protein bands relative to the corresponding β-tubulin band densities. Control values were normalized to 1 (mean ± SEM, n = 3; *p < 0.05, **p < 0.01, ***p < 0.001 compared with control; ^##^ *p* < 0.01, ^###^ *p* < 0.001 compared with VPA alone; *ns*, not significant). **(C)** Confocal microscopy images of cortical neurons from various experimental groups. Cells were cultured for 7 days, fixed, and subsequently immunostained for vGluT1 (red), with nuclei counterstained using DAPI (blue). Scale bar: 50 μm.

To elucidate the key role of specific cell types in regulating E/I balance within a VPA-induced ASD mouse model, cortical neurons and astrocytes were cultured either separately or together, and various E/I neuronal activities were assessed. Primary cortical neurons isolated from E15 mouse brains (Figure 7A) were stained for vGluT1, a vesicular glutamate transporter and a specific marker of glutamatergic neurons. In the VPA-treated group, vGluT1 expression was highly elevated (*p* ≤ 0.001); however, co-administration of 5 or 10 g/kg Gb extract restored vGluT1 levels to those observed in the control group (*p* ≤ 0.01) (Figure 7C). Further analysis of glutamatergic and GABAergic transporters, both pre- and postsynaptic, as well as vesicular transporters, was performed using western blotting (Figures 7B,D). In the VPA group, NMDA R1 expression, a receptor that amplifies excitatory synaptic activity, was reduced to approximately half of the control level (*p* ≤ 0.001). However, this expression was restored to control levels in both the 5 and 10 g/kg Gb extract-treated groups (5 g/kg, *NS*; 10 g/kg, *p* ≤ 0.001) (Figure 7B). GABA, the primary inhibitory neurotransmitter, and its excitatory counterpart, glutamate, were also analyzed. In the VPA group, GABA R1α expression was nearly doubled compared with the control group (*p* ≤ 0.001); however, treatment with 5 and 10 g/kg Gb extract restored its expression to control levels in a dose-dependent manner (*p* ≤ 0.001) (Figure 7B). Similarly, VGAT expression, which is associated with inhibitory neurotransmission, was significantly increased in the VPA group (*p* ≤ 0.001), but normalized in both the Gb extract-treated groups (*p* ≤ 0.001) (Figure 7B). In contrast, vGluT1 expression, which was significantly elevated in the VPA group (*p* ≤ 0.001), was restored to control levels following Gb extract treatment (*p* ≤ 0.01) (Figure 7B). Furthermore, GRM5 expression was nearly doubled in the VPA group compared to that in the control group (*p* ≤ 0.001) but was notably reduced in the Gb extract-treated groups, particularly at the 5 g/kg dose (5 g/kg, *p* ≤ 0.01; 10 g/kg, *p* ≤ 0.01) (Figure 7C). Expression of Tuj-1, a neuron-specific marker, was significantly reduced in the VPA group (*p* ≤ 0.001); however, it showed a substantial increase in the 5 g/kg Gb extract-treated group, surpassing control levels (*p* ≤ 0.001). In contrast, Tuj-1 expression in the 10 g/kg group was similar to that of the control (*p* ≤ 0.01) (Figure 7D). Both NLGN3 and NRXN1, presynaptic and postsynaptic proteins, respectively, were significantly reduced in the VPA group (*p* ≤ 0.001), and treatment with Gb extract almost completely restored these VPA-induced effects (5 g/kg, *p* ≤ 0.05 and *p* ≤ 0.01 respectively; 10 g/kg, *p* ≤ 0.05 and *p* ≤ 0.01, respectively) (Figure 7D).

**FIGURE 8:**
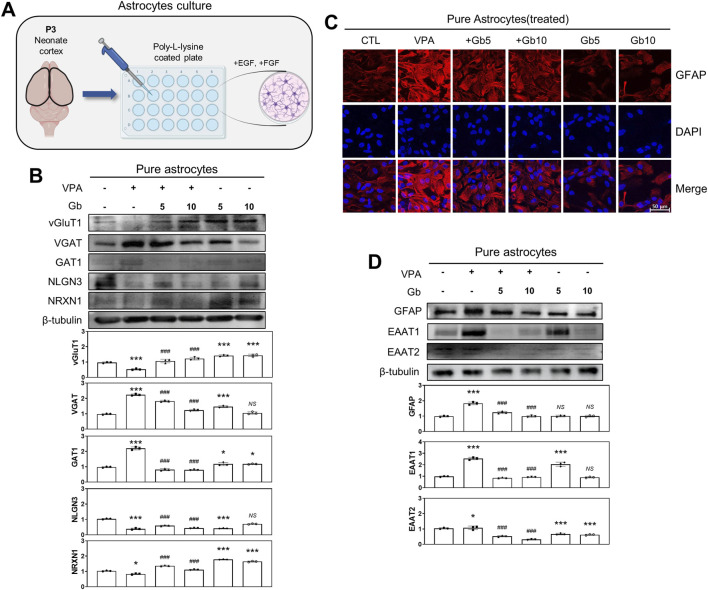
Regulatory effects of *Gryllus bimaculatus* (Gb) extract on excitatory and inhibitory neurotransporter activity in primary astrocytes from valproic acid (VPA)-treated postnatal mice. **(A)** Schematic representation of primary cortical astrocyte cultures derived from postnatal mouse brains. Experimental groups included CTL (saline, n = 8); VPA (600 mg/kg VPA, n = 8); VPA + Gb*5* (600 mg/kg VPA + 5 g/kg Gb extract, n = 8); VPA + Gb*10* (600 mg/kg VPA + 10 g/kg Gb extract, n = 8); Gb*5* (5 g/kg Gb extract, n = 8); Gb*10* (10 g/kg Gb extract, n = 8). **(B,D)** Western blot analysis of vGluT1, VGAT, GAT1, NLGN3, NRXN1, GFAP, EAAT1, and EAAT2 in astrocyte lysates. Equal amounts of protein were loaded per lane, with β-tubulin serving as the loading control. The bars represent fold-changes in the densitometric values of individual protein bands relative to β-tubulin densities. Control values were normalized to 1 (mean ± SEM, n = 3; ***p < 0.001 compared with control; ^###^ *p* < 0.001 compared with VPA alone; *ns*, not significant). **(C)** Confocal microscopy images of cortical astrocytes from the various experimental groups. Astrocytes were cultured as monolayers for 10 days, then fixed and immunostained for GFAP (red), with nuclei counterstained using DAPI (blue). Scale bar: 50 μm.

To investigate the role of cortical astrocytes in E/I balance regulation within the VPA-induced ASD mouse model, astrocytes were isolated from P3 mouse brains (Figure 8A), cultured, and immunostained. Immunofluorescence analysis of GFAP, an astrocyte-specific marker, revealed that GFAP expression was significantly elevated in the VPA group compared with the control group (Figure 8C). Treatment with 5 and 10 g/kg of Gb extract normalized GFAP expression to control levels. Notably, no significant difference was observed in GFAP expression between the control and Gb extract-only groups (Figure 8C). Western blot quantification further confirmed that GFAP expression in the VPA group was approximately twice that of the control group (*p* ≤ 0.001) (Figure 8D). However, administration of Gb extract at both 5 and 10 g/kg doses marked decreased GFAP expression compared with the VPA group (*p* ≤ 0.001). Regarding excitatory markers, vGluT1 expression was significantly reduced in the VPA group relative to the control group (*p* ≤ 0.001), but was restored following Gb extract treatment at both doses (5 and 10 g/kg, *p* ≤ 0.001). Conversely, VGAT levels, an inhibitory marker, were more than doubled in the VPA group than in the control group (*p* ≤ 0.001). Treatment with both 5 and 10 g/kg Gb extract significantly reduced VGAT expression, with greater reductions observed at the higher dose (*p* ≤ 0.001) (Figure 8B). Additionally, the expression of astrocyte-specific glutamate transporters, such as excitatory amino acid transporter 1/2 (EAAT1/2), was examined. Western blots analysis revealed that VPA treatment significantly increased EAAT1/2 expression (*p* ≤ 0.001 and *p* ≤ 0.05, respectively), which was reversed by combined treatment with Gb extract (*p* ≤ 0.001) (Figure 8D).

**FIGURE 9:**
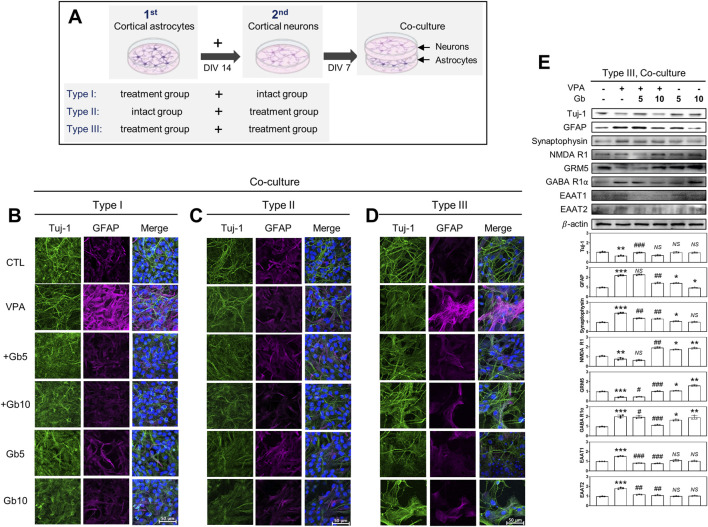
Crucial role of astrocytes in excitatory and inhibitory (E/I) neurotransporter activities in *Gryllus bimaculatus* (Gb) extract-treated mixed cultures from valproic acid (VPA)-treated mouse brain. **(A)** Schematic representation of three different types of mixed culture systems derived from embryonic and postnatal mouse brains: Type 1, astrocytes from each treatment group combined with neurons from untreated mice; Type 2, astrocytes from untreated mice combined with neurons from each treatment group; Type 3, astrocytes and neurons both derived from the same treatment group. Astrocytes from postnatal day 3 mouse brains were seeded for 7 days, followed by the addition of cortical neurons from embryonic day 15 mouse brains onto astrocytes monolayers for an additional 7 days. **(B–D)** Confocal microscopy images of the different types of mixed cultures. Cells were fixed and immunostained for Tuj-1 (green) and GFAP (purple), with nuclei counterstained using DAPI (blue). Scale bar: 50 μm. Experimental groups included CTL (saline, n = 8); VPA (600 mg/kg VPA, n = 8); VPA + Gb*5* (600 mg/kg VPA + 5 g/kg Gb extract, n = 8); VPA + Gb*10* (600 mg/kg VPA + 10 g/kg Gb extract, n = 8); Gb*5* (5 g/kg Gb extract, n = 8); Gb*10* (10 g/kg Gb extract, n = 8). **(E)** Western blots analysis of type III mixed culture. Cell lysates were immunoblotted for Tuj-1, GFAP, synaptophysin, NMDA receptor 1 (NMDA R1), GABA receptor 1α (GABA R1α), EAAT1, and EAAT2. Equal amounts of protein were loaded per each lane, with β-actin serving as the loading control. Bars represent fold-changes in the densitometric values of the bands relative to the corresponding β-actin densities. Control values were normalized to 1 (mean ± SEM, n = 3; *p < 0.05, **p < 0.01, ***p < 0.001 compared with control; ^#^ *p* < 0.05, ^##^ *p* < 0.01, ^###^ *p* < 0.001 compared with VPA alone; *ns*, not significant).

Mixed cultures were prepared using three different approaches, as illustrated in Figure 9A: Type I, neurons from age-matched intact ICR mice were cultured on astrocyte feeder cells derived from each treatment group; Type II, neurons from each treatment group were cultured on astrocyte feeder cells from untreated intact ICR mice; and Type III, neurons and astrocytes from the same treatment group were co-cultured.

Immunofluorescence microscopy and western blotting analyses were conducted under various mixed culture conditions to investigate the key cell types involved in the regulation of E/I balance and synaptic plasticity in a VPA-induced ASD mouse model. Type I, II, and III mixed cultures were used for western blotting. In both Type I and Type III mixed cultures, the VPA group exhibited a significant reduction in Tuj-1 expression, along with marked increase in GFAP and synaptophysin expression (*p* ≤ 0.01, *p* ≤ 0.001, *p* ≤ 0.001, respectively) (Figures 9B,D,E). Combined treatment with Gb extract at either 5 or 10 g/kg significantly restored the abnormal protein expression induced by VPA to control levels (5 g/kg, *p* ≤ 0.001, *NS*, *p* ≤ 0.01, respectively; 10 g/kg *NS*, *p* ≤ 0.01, *p* ≤ 0.01, respectively), whereas Gb extract alone did not affect the expression levels of Tuj-1, GFAP, or synaptophysin (Figures 9B,D,E). Notably, in the Type II mixed culture, which was created using astrocytes from the intact group and neurons from each treatment group, VPA treatment did not alter Tuj-1 or GFAP expression (Figure 9C).

Furthermore, the expression levels of key neurotransmission receptors, including NMDA R1, GABA R1α, and astrocytic glutamate transporters EAAT1 and EAAT2, were evaluated via western blotting (Figure 9E). Consistent with findings in pure neuron cultures, VPA treatment significantly elevated GABA R1α expression (*p* ≤ 0.001) (Figure 9E). However, this upregulation was dose-dependently suppressed by Gb extract, restoring expression to near-normal levels (5 g/kg, *p* ≤ 0.05; 10 g/kg, *p* ≤ 0.001). Similarly, NMDA R1 expression followed a comparable pattern to that observed in pure neuron cultures under VPA and Gb extract treatments (Figures 7C, 9E), although changes were less pronounced than those observed with GABA R1α (*p* ≤ 0.01). Additionally, the astrocytic glutamate transporters EAAT1 and EAAT2, which are essential for maintaining low extracellular glutamate levels, were markedly upregulated by VPA treatment (*p* ≤ 0.001) (Figure 9E). 5 g/kg or 10 g/kg of Gb extract with VPA 600 mg/kg effectively restored the expression of these transporters to control levels (5 g/kg, *p* ≤ 0.001 and *p* ≤ 0.001, respectively; 10 g/kg, *p* ≤ 0.01) (Figure 9E).

## Discussion

This study explicates the mechanisms by which Gb extract mitigates neural deficits in an ASD model, with a particular focus on its effects on impaired NPC proliferation, NPC differentiation, E/I imbalance, and abnormal synaptogenesis. Given the critical role of astrocytes in regulating E/I signaling, their contribution to the pathophysiology of ASD was examined. Gb extract, which contains approximately 61.3% protein, 13.4% fat, 10% fiber, and 3.9% ash (Hall et al., 2017), is well-known for its antioxidant and anti-inflammatory properties (Pedrosa et al., 2015). Although various biological benefits have been investigated, its specific neuroprotective functions and underlying mechanisms remain largely uncharacterized. This study demonstrates the prophylactic effects of Gb extract in an ASD animal model and elucidate astrocyte-associated molecular mechanisms.

Autism-like features were induced by administering VPA to pregnant mice, a widely used model for simulating ASD characteristics (Gzielo et al., 2020; Qi et al., 2022). Molecular analyses were conducted at E14, P3, and P40 to track age-dependent changes. Consistent with previous findings, NPCs from ASD model mice exhibited increased proliferation but impaired differentiation, which are hallmarks of abnormal brain growth driven by dysregulated PTEN and β-catenin signaling. Moreover, synaptic adhesion molecules, such as NRXNs and NLGNs, which are essential for synaptic connectivity, were significantly downregulated, thereby weakening presynaptic-postsynaptic interactions. Gb extract treatment effectively restored these deficits to near-control levels, highlighting its pronounced neuroprotective role.

The VPA-induced ASD model was further validated through altered ultrasonic vocalizations during the neonatal period (Gao et al., 2019), the emergence of anxiety-like behaviors at P32 (Wang et al., 2020), and the persistence of behavioral alterations into adulthood (Chen et al., 2015). Consistent with studies demonstrating that NPCs derived from human induced pluripotent stem cells of patients with ASD exhibit inherently higher proliferation, leading to abnormal brain growth driven by imbalanced PTEN and β-catenin signaling (Go et al., 2012), as well as findings from SHANK3-deficient mice (Wang et al., 2022), the present model accurately recapitulates key pathological features of ASD. Although the establishment of the ASD model in this study was supported by aberrant NPC proliferation and differentiation, and behavioral abnormalities had been confirmed in our previous work at a comparable developmental stage, the absence of behavioral evaluation herein constitutes a limitation.

The effects of both VPA and the Gb extract were assessed, and significant alterations were detected as early as E15. This rapid onset is particularly noteworthy given that the neuroprotective actions of the Gb extract have traditionally been attributed to its antioxidant and anti-inflammatory properties. However, emerging evidence indicates that Gb extract and other plant-derived bioactive compounds can rapidly modulate redox and inflammatory signaling networks through direct scavenging of reactive oxygen species, early suppression of microglial activation, and prompt upregulation of neurotrophic factors (Gupta et al., 2023; Singh et al., 2019). Such mechanisms are capable of initiating protective molecular responses within hours to days, consistent with the early alterations observed at E15 (Ghosh et al., 2016). These findings suggest that Gb extract may exert immediate neuromodulatory effects beyond its canonical antioxidant activity, potentially through modulation of intracellular signaling, regulation of synaptic protein dynamics, or activation of redox-sensitive transcriptional pathways that collectively contribute to the early onset of neuroprotection following VPA-induced developmental neurotoxicity.

In the context of synaptic communication, genetic alterations in NRXNs and NLGNs have been implicated in ASD and other cognitive disorders. The present analysis revealed a significant reduction in the expression of these proteins from E14 to P40 in the ASD mouse model; however, the decrease in NLGN2/3 expression was relatively minor at P40. These observations indicate that presynaptic-postsynaptic interactions are compromised, a notion supported by a previous study (Gogolla et al., 2009). Additionally, changes in dendritic spine density and excessive dendritic and synaptic genesis, as demonstrated by synaptophysin immunofluorescence staining, aligns with reports of abnormal brain growth during early development in ASD, which is often marked by increased head circumference (Lan et al., 2021; Zhang et al., 2019).

While the presence of Gb extract in maternal milk was not directly assessed in this study, the transfer of related insect-derived and natural products into breast milk has been documented in rodent models (Scarborough et at., 2021). The administration of Gb extract from birth to postnatal day 40 (P40) was intended to simulate continuous exposure across gestational, lactational, and early juvenile periods. This experimental paradigm is widely used in maternal-offspring studies to model real-world scenarios in which neonates are exposed to bioactive agents both prenatally via the placenta and postnatally via milk. Numerous reports have demonstrated the transfer of xenobiotics−including insecticides, persistent organic pollutants, and certain natural extracts− into rodent milk, leading to measurable physiological and behavioral alterations in nursing offspring (Burke et al., 2018). Postnatal exposure through maternal milk has also been shown to induce long-lasting changes in metabolism, physiology, and behavior that may differ from those elicited by prenatal exposure alone (Xie et al., 2018). Future studies should include pharmacokinetic analyses of Gb extract in maternal milk and experimental designs that distinguish between prenatal and postnatal exposure window to more clearly delineate the contribution of each developmental period.

The balance between excitation and inhibition is fundamental for homeostatic synaptic plasticity and optimal cortical network function (Lin et al., 2013). In the present study, reduced excitatory synaptic activity coupled with elevated inhibitory synaptic activity at E14 and P3 was marked by increased GABAergic neurotransmitter expression, reflecting a well-documented E/I imbalance in ASD models (Zhao et al., 2021). Although elevated NMDAR expression has been associated with early autism characteristics (Chow et al., 2012), the observed reduction in NMDAR expression in the VPA model suggests a potential compensatory mechanism to restore excitatory synapse functionality (Varghese et al., 2017). Moreover, distinct patterns of E/I synapse expression between the early (E14 and P3) and late (P40) developmental phases underscore the age-dependent variations in ASD pathology (Akitake et al., 2015). Importantly, treatment with Gb extract alleviated these aberrant protein expression levels, suggesting that maternal intake of Gb extract may help prevent the manifestation of ASD during both embryonic development and adolescence. However, this study does not include electrophysiological recordings, calcium imaging, or behavioral analyses; therefore, the functional consequences of these molecular changes remain uncertain.

To further elucidate the contributions of distinct cellular components, expression changes in receptors and vesicular proteins regulating E/I neurotransmission were examined in the cortical tissues of ASD model mice, along with separate neuron and astrocyte cultures. The findings highlight the critical role of astrocytes not only in buffering and clearing glutamate and GABA, the primary E/I neurotransmitters of the brain, respectively, but also in regulating neuronal activity and synaptic plasticity. In pure neuronal cultures, VPA treatment increased the expression of vGluT1 in presynaptic neurons while reducing NMDA R1 expression in postsynaptic neurons and markedly upregulating mGluR5. Concurrently, VPA treatment significantly increased VGAT expression in cortical astrocytes and suppressed the expression of the synaptic adhesion molecules NRXN1 and NLGN3, suggesting that excessive accumulation of glutamate in presynaptic vesicles, predominantly released via mGluR5 in postsynaptic neurons, may be counterbalanced by enhanced GABA loading and release (Zhao et al., 2021).

Astrocytes play a crucial role in maintaining homeostasis of the central nervous system, supporting neuronal function, and regulating glutamate and Ca^2+^ signaling. Consistent with previous reports of increased GFAP expression in various brain regions of individuals with autism (Vakilzadeh et al., 2022), cortical astrocytes from VPA-exposed ASD mice exhibited marked GFAP upregulation, indicative of astrocyte activation. This activation likely reflects neural damage, inflammation, or developmental abnormalities (Xiong et at., 2023). Furthermore, the reduced expression of astrocytic vGluT1 suggests a diminished capacity for glutamate release, whereas upregulated excitatory amino acid transporters (EAAT1 and EAAT2) may represent a compensatory mechanism to prevent excitation, a phenomenon also linked to neuronal loss in various neuropathologies (Edmonson et al., 2014; Parkin et al., 2020; Takeda et al., 2021).

Neuron-astrocyte co-culture experiments further revealed that astrocytes from VPA-exposed mice exhibited significantly increased GFAP expression, whereas Tuj-1 expression, an indicator of neuronal differentiation, was suppressed exclusively when both neurons and astrocytes originated from VPA-treated mice. These results underscore the pivotal role of astrocytes in modulating neuronal differentiation and function in patients with ASD.

A limitation of this study lies in the inability to precisely quantify the expression levels of excitatory- and inhibitory-related proteins corresponding to Types I, II, and III within the astrocyte–neuron co-culture system. While these co-culture experiments provide evidence suggestive of astrocyte-driven modulation of neuronal differentiation and synaptic protein expression, the inability to clearly disentangle the respective contributions of neurons and astrocytes constrains the strength of the mechanistic conclusions.

Accordingly, these findings should not be overstated as definitive proof of astrocyte involvement without additional corroborative data. Nevertheless, the critical role of astrocytes in neural development and synaptic function is strongly supported by prior research. To further substantiate the functional relevance of astrocyte regulation, future investigations will incorporate electrophysiological approaches such as patch-clamp recordings and multi-electrode array analyses, as well as *in vivo* imaging techniques including calcium imaging and voltage-sensitive dye imaging of neuronal activity.

## Conclusion

These findings reinforce the pivotal role of astrocytes in the pathogenesis of ASD, particularly in the regulation of E/I neurotransmission balance. The neuroprotective effects observed in this study were primarily mediated through astrocyte-driven modulation of synaptic activity. Primary culture experiments further emphasized the central role of astrocytes in maintaining neural homeostasis, with Gb extract serving as a useful tool to prove glial function. The effects of Gb extract on the protein-level excitatory/inhibitory balance and cellular changes were confirmed. However, to determine whether the protein-level recovery induced by Gb extract, as well as synaptic transmission and circuit-level activity, ultimately lead to functional recovery in ASD, further evaluations including behavioral and electrophysiological assessments are necessary. Taken together, these findings identify astrocytes as potential key therapeutic targets in ASD and underscore the promise of edible insect-derived compounds as modulators of glial activity. This work contributes to the broader exploration of astrocyte-based interventions for neurodevelopmental and psychiatric disorders, laying a foundation for novel preventive and therapeutic strategies.

## Data Availability

The original contributions presented in the study are included in the article/Supplementary Material, further inquiries can be directed to the corresponding author.

## References

[B1] Abhishek M. Rubal S. Rohit K. Rupa J. Phulen S. Gurjeet K. (2022). Neuroprotective effect of the standardised extract of Bacopa monnieri (BacoMind) in valproic acid model of autism spectrum disorder in rats. J. Ethnopharmacol. 293, 115199. 10.1016/j.jep.2022.115199 35346813

[B2] Ahn M. Y. Han J. W. Hwang J. S. Yun E. Y. Lee B. M. (2014). Anti-inflammatory effect of glycosaminoglycan derived from gryllus bimaculatus (a type of cricket, insect) on adjuvant-treated chronic arthritis rat model. J. Toxicol. Environ. Health A. 77 (22-24), 1332–1345. 10.1080/15287394.2014.951591 25343284

[B3] Akitake Y. Katsuragi S. Hosokawa M. Mishima K. Ikeda T. Miyazato M. (2015). Moderate maternal food restriction in mice impairs physical growth, behavior, and neurodevelopment of offspring. Nutr. Res. 35 (1), 76–87. 10.1016/j.nutres.2014.10.014 25433908

[B5] Bhandari R. Kuhad A. (2015). Neuropsychopharmacotherapeutic efficacy of curcumin in experimental paradigm of autism spectrum disorders. Life. Sci. 141, 156–169. 10.1016/j.lfs.2015.09.012 26407474

[B6] Bi X. A. Liu Y. Jiang Q. Shu Q. Sun Q. Dai J. (2018). The diagnosis of autism spectrum disorder based on the random neural network cluster. Front. Hum. Neurosci. 12, 257. 10.3389/fnhum.2018.00257 29997489 PMC6028564

[B8] Burke A. P. Niibori Y. Terayama H. Ito M. Pidgeon C. Arsenault J. (2018). Mammalian susceptibility to a neonicotinoid Insecticide after fetal and early postnatal exposure. Sci. Rep. 8 (1), 16639. 10.1038/s41598-018-35129-5 30413779 PMC6226530

[B9] Buu Tran N. Lee H. Lee S.-J. (2024). Protective effects of *Tenebrio molitor* and Protaetia brevitarsis seulensis extracts on aging: regulation of blood–brain barrier, amyloid β plaques, and intestinal inflammation in D-galactose-induced aging mice. J. Funct. Foods. 119, 106333. 10.1016/j.jff.2024.106333

[B10] Chaste P. Leboyer M. (2012). Autism risk factors: genes, environment, and gene-environment interactions. Dialogues. Clin. Neurosci. 14 (3), 281–292. 10.31887/DCNS.2012.14.3/pchaste 23226953 PMC3513682

[B11] Chateauvieux S. Morceau F. Dicato M. Diederich M. (2010). Molecular and therapeutic potential and toxicity of valproic acid. J. Biomed. Biotechnol. 2010, 479364. 10.1155/2010/479364 20798865 PMC2926634

[B12] Chen Y. Huang W. C. Sejourne J. Clipperton-Allen A. E. Page D. T. (2015). Pten mutations alter brain growth trajectory and allocation of cell types through elevated beta-catenin signaling. J. Neurosci. 35 (28), 10252–10267. 10.1523/JNEUROSCI.5272-14.2015 26180201 PMC6605343

[B77] Choi D. W. Maulucci-Gedde M. Kriegstein A. R. (1987). Glutamate neurotoxicity in cortical cell culture. J. Neurosci. 7 (2), 357–368. 10.1523/JNEUROSCI.07-02-00357.1987 2880937 PMC6568898

[B13] Chow M. L. Pramparo T. Winn M. E. Barnes C. C. Li H. R. Weiss L. (2012). Age-dependent brain gene expression and copy number anomalies in autism suggest distinct pathological processes at young *versus* mature ages. PLoS Genet. 8 (3), e1002592. 10.1371/journal.pgen.1002592 22457638 PMC3310790

[B14] Cruz-Martins N. Quispe C. Kirkin C. Senol E. Zulug A. Özçelik B. (2021). Paving plant-food-derived bioactives as effective therapeutic agents in autism spectrum disorder. Oxid. Med. Cell. Longev. 2021, 1131280. 10.1155/2021/1131280 34471461 PMC8405324

[B16] Deckmann I. Schwingel G. B. Fontes-Dutra M. Bambini-Junior V. Gottfried C. (2018). Neuroimmune alterations in autism: a translational analysis focusing on the animal model of autism induced by prenatal exposure to valproic acid. Neuroimmunomodulation 25 (5-6), 285–299. 10.1159/000492113 30157484

[B17] Edmonson C. Ziats M. N. Rennert O. M. (2014). Altered glial marker expression in autistic post-mortem prefrontal cortex and cerebellum. Mol. Autism 5 (1), 3. 10.1186/2040-2392-5-3 24410870 PMC3914711

[B18] Eissa N. Jayaprakash P. Azimullah S. Ojha S. K. Al-Houqani M. Jalal F. Y. (2018). The histamine H3R antagonist DL77 attenuates autistic behaviors in a prenatal valproic acid-induced mouse model of autism. Sci. Rep. 8 (1), 13077. 10.1038/s41598-018-31385-7 30166610 PMC6117350

[B20] Gao X. Zheng R. Ma X. Gong Z. Xia D. Zhou Q. (2019). Elevated level of PKMζ underlies the excessive anxiety in an autism model. Front. Mol. Neurosci. 12, 291. 10.3389/fnmol.2019.00291 31849605 PMC6893886

[B21] Ghosh A. Langley M. R. Harischandra D. S. Neal M. L. Jin H. Anantharam V. (2016). Mitoapocynin treatment protects against neuroinflammation and dopaminergic neurodegeneration in a preclinical animal model of parkinson's disease. J. Neuroimmune. Pharmacol. 11 (2), 259–278. 10.1007/s11481-016-9650-4 26838361 PMC4995106

[B22] Gilbert J. Man H. Y. (2017). Fundamental elements in autism: from neurogenesis and neurite growth to synaptic plasticity. Front. Cell. Neurosci. 11, 359. 10.3389/fncel.2017.00359 29209173 PMC5701944

[B23] Go H. S. Seo J. E. Kim K. C. Han S. M. Kim P. Kang Y. S. (2011). Valproic acid inhibits neural progenitor cell death by activation of NF-κB signaling pathway and up-regulation of Bcl-XL. J. Biomed. Sci. 18 (1), 48. 10.1186/1423-0127-18-48 21722408 PMC3158748

[B24] Go H. S. Kim K. C. Choi C. S. Jeon S. J. Kwon K. J. Han S. H. (2012). Prenatal exposure to valproic acid increases the neural progenitor cell pool and induces macrocephaly in rat brain *via* a mechanism involving the GSK-3β/β-catenin pathway. Neuropharmacology 63 (6), 1028–1041. 10.1016/j.neuropharm.2012.07.028 22841957

[B25] Gogolla N. Leblanc J. J. Quast K. B. Sudhof T. C. Fagiolini M. Hensch T. K. (2009). Common circuit defect of excitatory-inhibitory balance in mouse models of autism. J. Neurodev. Disord. 1 (2), 172–181. 10.1007/s11689-009-9023-x 20664807 PMC2906812

[B26] Guang S. Pang N. Deng X. Yang L. He F. Wu L. (2018). Synaptopathology involved in autism spectrum disorder. Front. Cell. Neurosci. 12, 470. 10.3389/fncel.2018.00470 30627085 PMC6309163

[B27] Gupta S. Ellis S. E. Ashar F. N. Moes A. Bader J. S. Zhan J. (2014). Transcriptome analysis reveals dysregulation of innate immune response genes and neuronal activity-dependent genes in autism. Nat. Commun. 5, 5748. 10.1038/ncomms6748 25494366 PMC4270294

[B28] Gupta D. P. Park S. H. Lee Y. S. Lee S. Lim S. Byun J. (2023). Daphne genkwa flower extract promotes the neuroprotective effects of microglia. Phytomedicine 108, 154486. 10.1016/j.phymed.2022.154486 36240609

[B29] Gzielo K. Soltys Z. Rajfur Z. Setkowicz Z. K. (2019). The impact of the ketogenic diet on glial cells morphology. A quantitative morphological analysis. Neurosci 413, 239–251. 10.1016/j.neuroscience.2019.06.009 31220541

[B30] Gzielo K. Potasiewicz A. Holuj M. Litwa E. Popik P. Nikiforuk A. (2020). Valproic acid exposure impairs ultrasonic communication in infant, adolescent and adult rats. Eur. Neuropsychopharmacol. 41, 52–62. 10.1016/j.euroneuro.2020.09.006 32978035

[B31] Hall F. G. Jones O. G. O'Haire M. E. Liceaga A. M. (2017). Functional properties of tropical banded cricket (Gryllodes sigillatus) protein hydrolysates. Food. Chem. 224, 414–422. 10.1016/j.foodchem.2016.11.138 28159288

[B32] Horder J. Petrinovic M. M. Mendez M. A. Bruns A. Takumi T. Spooren W. (2018). Glutamate and GABA in autism spectrum disorder-a translational magnetic resonance spectroscopy study in man and rodent models. Transl. Psychiatry. 8 (1), 106. 10.1038/s41398-018-0155-1 29802263 PMC5970172

[B34] Hsiao E. Y. McBride S. W. Hsien S. Sharon G. Hyde E. R. McCue T. (2013). Microbiota modulate behavioral and physiological abnormalities associated with neurodevelopmental disorders. Cell 155 (7), 1451–1463. 10.1016/j.cell.2013.11.024 24315484 PMC3897394

[B35] Hutton S. R. Pevny L. H. (2008). Isolation, culture, and differentiation of progenitor cells from the central nervous system. *CSH. Protoc.* 2008, pdb Prot. 2008, 5077. 10.1101/pdb.prot5077 21356718

[B36] Hwang B. B. Chang M. H. Lee J. H. Heo W. Kim J. K. Pan J. H. (2019). The edible insect gryllus bimaculatus protects against gut-derived inflammatory responses and liver damage in mice after acute alcohol exposure. Nutrients 11 (4), 857. 10.3390/nu11040857 30995745 PMC6521266

[B38] Kim K. C. Lee D. K. Go H. S. Kim P. Choi C. S. Kim J. W. (2014). Pax6-dependent cortical glutamatergic neuronal differentiation regulates autism-like behavior in prenatally valproic acid-exposed rat offspring. Mol. Neurobiol. 49 (1), 512–528. 10.1007/s12035-013-8535-2 24030726

[B39] Kumamaru E. Egashira Y. Takenaka R. Takamori S. (2014). Valproic acid selectively suppresses the formation of inhibitory synapses in cultured cortical neurons. Neurosci. Lett. 569, 142–147. 10.1016/j.neulet.2014.03.066 24708928

[B40] Lan J. Hu Y. Wang X. Zheng W. Liao A. Wang S. (2021). Abnormal spatiotemporal expression pattern of progranulin and neurodevelopment impairment in VPA-induced ASD rat model. Neuropharmacology 196, 108689. 10.1016/j.neuropharm.2021.108689 34175324

[B41] Lenart J. Augustyniak J. Lazarewicz J. W. Zieminska E. (2020). Altered expression of glutamatergic and GABAergic genes in the valproic acid-induced rat model of autism: a screening test. Toxicol 440, 152500. 10.1016/j.tox.2020.152500 32428529

[B42] Lin H. C. Gean P. W. Wang C. C. Chan Y. H. Chen P. S. (2013). The amygdala excitatory/inhibitory balance in a valproate-induced rat autism model. PLoS One 8 (1), e55248. 10.1371/journal.pone.0055248 23383124 PMC3558482

[B43] Lord C. Elsabbagh M. Baird G. Veenstra-Vanderweele J. (2018). Autism spectrum disorder. Lancet 392 (10146), 508–520. 10.1016/S0140-6736(18)31129-2 30078460 PMC7398158

[B44] Masi A. DeMayo M. M. Glozier N. Guastella A. J. (2017). An overview of autism spectrum disorder, heterogeneity and treatment options. Neurosci. Bull. 33 (2), 183–193. 10.1007/s12264-017-0100-y 28213805 PMC5360849

[B78] McCarthy K. D. de Vellis J. (1980). Preparation of separate astroglial and oligodendroglial cell cultures from rat cerebral tissue. J. Cell. Biol. 85 (3), 890–902. 10.1083/jcb.85.3.890 6248568 PMC2111442

[B45] McPheeters M. L. Warren Z. Sathe N. Bruzek J. L. Krishnaswami S. Jerome R. N. (2011). A systematic review of medical treatments for children with autism spectrum disorders. Peds 127 (5), e1312–e1321. 10.1542/peds.2011-0427 21464191

[B46] Morrison-Levy N. Go C. Ochi A. Otsubo H. Drake J. Rutka J. (2018). Children with autism spectrum disorders and drug-resistant epilepsy can benefit from epilepsy surgery. Epilepsy. Behav. 85, 200–204. 10.1016/j.yebeh.2018.06.023 30032808

[B47] Owen R. Sikich L. Marcus R. N. Corey-Lisle P. Manos G. McQuade R. D. (2009). Aripiprazole in the treatment of irritability in children and adolescents with autistic disorder. Peds 124 (6), 1533–1540. 10.1542/peds.2008-3782 19948625

[B48] Park S. A. Lee G. H. Lee H. Y. Hoang T. H. Chae H. J. (2020). Glucose-lowering effect of gryllus bimaculatus powder on streptozotocin-induced diabetes through the AKT/mTOR pathway. Food. Sci. Nutr. 8 (1), 402–409. 10.1002/fsn3.1323 31993166 PMC6977414

[B49] Parkin G. M. Gibbons A. Udawela M. Dean B. (2020). Excitatory amino acid transporter (EAAT)1 and EAAT2 mRNA levels are altered in the prefrontal cortex of subjects with schizophrenia. J. Psychiatr. Res. 123, 151–158. 10.1016/j.jpsychires.2020.02.004 32065951

[B50] Pedrosa M. Boyano-Martinez T. Garcia-Ara C. Quirce S. (2015). Shellfish allergy: a comprehensive review. Clin. Rev. Allergy. Immunol. 49 (2), 203–216. 10.1007/s12016-014-8429-8 24870065

[B51] Qi C. Chen A. Mao H. Hu E. Ge J. Ma G. (2022). Excitatory and inhibitory synaptic imbalance caused by brain-derived neurotrophic factor deficits during development in a valproic acid mouse model of autism. Front. Mol. Neurosci. 15, 860275. 10.3389/fnmol.2022.860275 35465089 PMC9019547

[B52] Qiu J. Guo H. Li L. Xu Z. Xu Z. Jing X. (2020). Valproic acid therapy decreases serum 25-hydroxyvitamin D level in female infants and toddlers with epilepsy-a pilot longitudinal study. J. Biomed. Res. 35 (1), 61–67. 10.7555/JBR.34.20200057 33342771 PMC7874269

[B53] Romoli M. Mazzocchetti P. D'Alonzo R. Siliquini S. Rinaldi V. E. Verrotti A. (2019). Valproic acid and epilepsy: from molecular mechanisms to clinical evidences. Curr. Neuropharmacol. 17 (10), 926–946. 10.2174/1570159X17666181227165722 30592252 PMC7052829

[B54] Rumpold B. A. Schluter O. K. (2013). Nutritional composition and safety aspects of edible insects. Mol. Nutr. Food. Res. 57 (5), 802–823. 10.1002/mnfr.201200735 23471778

[B55] Russo A. J. (2009). Decreased serum Ou/Zn sOD in children with autism. *Nutr. Metab.* Insights. 2, NMI.S3733. 10.4137/nmi.S3733

[B56] Scarborough J. Mueller F. S. Weber-Stadlbauer U. Mattei D. Opitz L. Cattaneo A. (2021). A novel murine model to study the impact of maternal depression and antidepressant treatment on biobehavioral functions in the offspring. Mol. Psychiatry 26 (11), 6756–6772. 10.1038/s41380-021-01145-7 34002019 PMC8760069

[B58] Singh S. K. Srivastav S. Castellani R. J. Plascencia-Villa G. Perry G. (2019). Neuroprotective and antioxidant effect of Ginkgo biloba extract against AD and other neurological disorders. Neurotherapeutics 16 (3), 666–674. 10.1007/s13311-019-00767-8 31376068 PMC6694352

[B59] Takeda K. Watanabe T. Oyabu K. Tsukamoto S. Oba Y. Nakano T. (2021). Valproic acid-exposed astrocytes impair inhibitory synapse formation and function. Sci. Rep. 11 (1), 23. 10.1038/s41598-020-79520-7 33420078 PMC7794250

[B60] Talebizadeh Z. Bittel D. C. Veatch O. J. Butler M. G. Takahashi T. N. Miles J. H. (2004). Do known mutations in neuroligin genes (NLGN3 and NLGN4) cause autism? J. Autism. Dev. Disord. 34 (6), 735–736. 10.1007/s10803-004-5295-x 15679194 PMC5161028

[B62] Tran N. B. Lee S. J. (2023b). Effects of gryllus bimaculatus and Oxya chinensis sinuosa extracts on brain damage *via* blood-brain barrier control and apoptosis in mice with pentylenetetrazol-induced epilepsy. PLoS. One. 18 (9), e0291191. 10.1371/journal.pone.0291191 37695764 PMC10495007

[B63] Tran N. B. Lee H. Lee S.-J. (2023a). Extracts from the edible insects gryllus bimaculatus and Oxya chinensis sinuosa as an effective postnatal therapy for improving autistic behavior through blood-brain barrier control and gut microbiota. J. Funct. Foods. 104, 105516. 10.1016/j.jff.2023.105516

[B64] Vakilzadeh G. Falcone C. Dufour B. Hong T. Noctor S. C. Martinez-Cerdeno V. (2022). Decreased number and increased activation state of astrocytes in gray and white matter of the prefrontal cortex in autism. Cereb. Cortex 32 (21), 4902–4912. 10.1093/cercor/bhab523 35212358 PMC9627019

[B65] Varghese M. Keshav N. Jacot-Descombes S. Warda T. Wicinski B. Dickstein D. L. (2017). Autism spectrum disorder: neuropathology and animal models. Acta. Neuropathol. 134 (4), 537–566. 10.1007/s00401-017-1736-4 28584888 PMC5693718

[B66] Verkhratsky A. Nedergaard M. (2018). Physiology of astroglia. Physiol. Rev. 98 (1), 239–389. 10.1152/physrev.00042.2016 29351512 PMC6050349

[B67] Wang M. Wei P. C. Lim C. K. Gallina I. S. Marshall S. Marchetto M. C. (2020). Increased neural progenitor proliferation in a hiPSC model of autism induces replication stress-associated genome instability. Cell. Stem. Cell. 26 (2), 221–233.e6. 10.1016/j.stem.2019.12.013 32004479 PMC7175642

[B68] Wang Y. Chiola S. Yang G. Russell C. Armstrong C. J. Wu Y. (2022). Modeling human telencephalic development and autism-associated SHANK3 deficiency using organoids generated from single neural rosettes. Nat. Commun. 13 (1), 5688. 10.1038/s41467-022-33364-z 36202854 PMC9537523

[B69] Xie Y. Zeng H. Huang Z. Xu H. Fan Q. Zhang Y. (2018). Effect of maternal administration of edible bird's nest on the learning and memory abilities of suckling offspring in mice. Neural Plast. 2018, 7697261. 10.1155/2018/7697261 29765403 PMC5885349

[B71] Xiong Y. Chen J. Li Y. (2023). Microglia and astrocytes underlie neuroinflammation and synaptic susceptibility in autism spectrum disorder. Front. Neurosci. 17, 1125428. 10.3389/fnins.2023.1125428 37021129 PMC10067592

[B72] Yan J. Oliveira G. Coutinho A. Yang C. Feng J. Katz C. (2005). Analysis of the neuroligin 3 and 4 genes in autism and other neuropsychiatric patients. Mol. Psychiatry. 10 (4), 329–332. 10.1038/sj.mp.4001629 15622415

[B73] Yu S. H. Yu S. Y. Lee B. S. Kim H. J. Kim M. R. Lee Y. C. (2020). 28-day repeated oral dose toxicity study of an aqueous extract of gryllus bimaculatus in sprague-dawley rat. Toxicol. Rep. 7, 577–582. 10.1016/j.toxrep.2020.04.006 32426238 PMC7225595

[B74] Zablotsky B. Black L. I. Maenner M. J. Schieve L. A. Blumberg S. J. (2015). Estimated prevalence of autism and other developmental disabilities following questionnaire changes in the 2014 national health interview survey. Natl. Health. Stat. Rep. 87, 1–20. 26632847

[B75] Zhang Y. Xiang Z. Jia Y. He X. Wang L. Cui W. (2019). The notch signaling pathway inhibitor dapt alleviates autism-like behavior, autophagy and dendritic spine density abnormalities in a valproic acid-induced animal model of autism. Prog. Neuropsychopharmacol. Biol. Psychiatry. 94, 109644. 10.1016/j.pnpbp.2019.109644 31075347

[B76] Zhao H. Mao X. Zhu C. Zou X. Peng F. Yang W. (2021). GABAergic system dysfunction in autism spectrum disorders. Front. Cell. Dev. Biol. 9, 781327. 10.3389/fcell.2021.781327 35198562 PMC8858939

