# Supplementary material for: Astrocytic gatekeeping of neural circuitry and synaptic balance in an autism mouse model: mechanistic insights beyond Gryllus bimaculatus extract-derived therapy
Source: Front Cell Dev Biol. 2025 Nov 24;13:1677851. doi: 10.3389/fcell.2025.1677851 (PMC12682880; doi:10.3389/fcell.2025.1677851)
Supplement: Supplementary file 1 [file Table1.docx]

**Supplementary Table 1. Gb extract ES- total compound anaylsis results ( 50 of 163 components )**

| # | **Component name** | **Total**  **Fragments Found** | **Formula** | **Observed neutral mass**  **(Da)** | **Observed**  **m/z** | **Observed RT**  **(min)** | **Detector**  **counts** |
| --- | --- | --- | --- | --- | --- | --- | --- |
| 1 | 6-Isoinosine | 8 | C_10_H_12_N_4_O_5_ | 268.0799 | 267.0726 | 1.74 | 120983 |
| 2 | Methyl propyl disulfide | 0 | C_4_H_10_S_2_ | 122.0219 | 167.0201 | 0.92 | 120617 |
| 3 | Glutathione oxidized | 56 | C_20_H_32_N_6_O_12_S_2_ | 612.1519 | 611.1447 | 1.05 | 51980 |
| 4 | Sanleng acid | 14 | C_18_H_34_O_5_ | 330.2396 | 329.2323 | 12.69 | 46247 |
| 5 | Citric acid | 3 | C_6_H_8_O_7_ | 192.0261 | 191.0189 | 0.97 | 39795 |
| 6 | D-pantothenic acid | 3 | C_9_H_17_NO_5_ | 219.11 | 218.1028 | 2.99 | 25693 |
| 7 | Sanleng acid | 24 | C_18_H_34_O_5_ | 330.2397 | 329.2324 | 12.82 | 21591 |
| 8 | Xanthosine | 5 | C_10_H_12_N_4_O_6_ | 284.0743 | 283.067 | 2.39 | 19493 |
| 9 | Uridine | 12 | C_9_H_12_N_2_O_6_ | 244.0685 | 243.0612 | 1.2 | 18193 |
| 10 | Noroxyhydrastinine | 7 | C_10_H_9_NO_3_ | 191.0575 | 190.0502 | 6.65 | 17939 |
| 11 | 4-Hydroxyquinoline | 0 | C_9_H_7_NO | 145.0519 | 190.0501 | 6.41 | 16465 |
| 12 | Noroxyhydrastinine | 5 | C_10_H_9_NO_3_ | 191.0574 | 190.0501 | 6.41 | 16465 |
| 13 | Phloyoside Ⅱ | 4 | C_17_H_25_ClO_12_ | 456.1035 | 455.0962 | 4.74 | 14911 |
| 14 | Adenosine diphosphate | 10 | C_10_H_15_N_5_O_10_P_2_ | 427.0281 | 426.0208 | 0.72 | 14506 |
| 15 | Adenosine | 8 | C_10_H_13_N_5_O_4_ | 267.0952 | 312.0934 | 1.49 | 12084 |
| 16 | Methyl propyl disulfide | 0 | C_4_H_10_S_2_ | 122.0219 | 167.0201 | 1.23 | 10694 |
| 17 | Daturametelin A | 0 | C_34_H_48_O_9_ | 600.3271 | 599.3199 | 21.14 | 8876 |
| 18 | Riboflavin (Vitamin B2) | 10 | C_17_H_20_N_4_O_6_ | 376.1366 | 375.1294 | 5.18 | 8249 |
| 19 | Malvalic acid | 0 | C_18_H_32_O_2_ | 280.2407 | 279.2335 | 21.43 | 8016 |
| 20 | Sanleng acid | 0 | C_18_H_34_O_5_ | 330.2397 | 329.2324 | 10.72 | 7951 |
| 21 | Palmitoleic acid methyl ester | 4 | C_17_H_32_O_2_ | 268.2389 | 313.2371 | 14.26 | 7631 |
| 22 | Pantothenic acid | 5 | C_11_H_21_NO_5_ | 247.1409 | 292.1391 | 1.47 | 7412 |
| 23 | DL-Tyrosine | 2 | C_9_H_11_NO_3_ | 181.0731 | 180.0658 | 3.55 | 7067 |
| 24 | Dibutyl sebacate | 0 | C_18_H_34_O_4_ | 314.2449 | 313.2376 | 17.53 | 6916 |
| 25 | Ala | 0 | C_3_H_7_NO_2_ | 89.0481 | 134.0463 | 1.49 | 6437 |
| 26 | 9,12-Dihydroxy-15-nonadecenoic acid | 1 | C_19_H_36_O_4_ | 328.2601 | 373.2583 | 12.6 | 5733 |
| 27 | Gamma-tocotrienol | 1 | C_28_H_42_O_2_ | 410.3174 | 409.3101 | 25.21 | 5420 |
| 28 | Cys | 0 | C_3_H_7_NO_2_S | 121.0191 | 166.0174 | 0.74 | 5227 |
| 29 | Malic acid | 0 | C_4_H_6_O_5_ | 134.0221 | 179.0203 | 1.34 | 5214 |
| 30 | Hookeroside D | 17 | C_43_H_70_O_18_ | 874.456 | 873.4487 | 4.48 | 5195 |
| 31 | Cyclo(Pro-Val) | 3 | C_10_H_16_N_2_O_2_ | 196.1202 | 241.1184 | 5.22 | 4962 |
| 32 | Trifloroside | 3 | C_35_H_42_O_20_ | 782.2251 | 827.2233 | 0.74 | 4601 |
| 33 | 9,12-Dihydroxy-15-nonadecenoic acid | 7 | C_19_H_36_O_4_ | 328.2604 | 373.2586 | 12.73 | 4420 |
| 34 | (E)-9-Octadecenoic acid | 0 | C_18_H_34_O_2_ | 282.2547 | 281.2474 | 22.82 | 4372 |
| 35 | Coronaric acid | 3 | C_18_H_32_O_3_ | 296.234 | 295.2267 | 16.65 | 4090 |
| 36 | D-(+)-Trehalose | 8 | C_12_H_22_O_11_ | 342.1146 | 387.1128 | 0.62 | 4071 |
| 37 | Hypericin | 0 | C_30_H_16_O_8_ | 504.0836 | 503.0763 | 0.92 | 3954 |
| 38 | Methyl myristate | 2 | C_15_H_30_O_2_ | 242.2238 | 287.222 | 13.2 | 3897 |
| 39 | 2',3',4'-Trimethoxyaceto-phenone | 0 | C_11_H_14_O_4_ | 210.0896 | 255.0878 | 5.19 | 3776 |
| 40 | Gentianadine | 5 | C_8_H_7_NO_2_ | 149.047 | 148.0398 | 6.65 | 3698 |
| 41 | Dihydroxy stearic acid | 6 | C_18_H_36_O_4_ | 316.2604 | 315.2531 | 15.5 | 3600 |
| 42 | Cireneol G | 1 | C_17_H_30_O_2_ | 266.223 | 311.2213 | 14.77 | 3541 |
| 43 | Glycoside K | 15 | C_40_H_66_O_16_ | 802.4364 | 847.4346 | 5.59 | 3516 |
| 44 | Methyl heptadecanoate | 0 | C_18_H_36_O_2_ | 284.271 | 283.2637 | 24.27 | 3080 |
| 45 | Daturametelin A | 0 | C_34_H_48_O_9_ | 600.3284 | 599.3211 | 21.04 | 3039 |
| 46 | 4-Hydroxyquinoline | 0 | C_9_H_7_NO | 145.052 | 190.0502 | 8.04 | 3037 |
| 47 | Noroxyhydrastinine | 1 | C_10_H_9_NO_3_ | 191.0575 | 190.0502 | 8.04 | 3037 |
| 48 | Arg | 0 | C_6_H_14_N_4_O_2_ | 174.111 | 173.1038 | 0.58 | 2933 |
| 49 | DL-Arginine | 3 | C_6_H_14_N_4_O_2_ | 174.111 | 173.1038 | 0.58 | 2933 |
| 50 | Cireneol G | 2 | C_17_H_30_O_2_ | 266.2235 | 311.2217 | 13.37 | 2873 |

**Supplementary Table 2. Gb extract ES+ total compound anaylsis results ( 50 of 147 components )**

| **#** | **Component name** | **Total**  **Fragments Found** | **Formula** | **Observed neutral mass**  **(Da)** | **Observed**  **m/z** | **Observed RT**  **(min)** | **Detector**  **counts** |
| --- | --- | --- | --- | --- | --- | --- | --- |
| 1 | Adenosine | 5 | C_10_H_13_N_5_O_4_ | 267.0968 | 268.104 | 1.49 | 75758 |
| 2 | L-Arginyl-N-2-naphthalenyl-L-argininamide | 4 | C_22_H_33_N_9_O_2_ | 455.2747 | 456.282 | 4.19 | 60035 |
| 3 | Adenine | 3 | C_5_H_5_N_5_ | 135.0548 | 136.0621 | 1.49 | 31649 |
| 4 | Hypoxanthine | 2 | C_5_H_4_N_4_O | 136.0386 | 137.0458 | 1.74 | 23692 |
| 5 | E-p-Coumatic acid | 5 | C_9_H_8_O_3_ | 164.0474 | 165.0547 | 1.21 | 23205 |
| 6 | o-Coumaric acid | 5 | C_9_H_8_O_3_ | 164.0474 | 165.0547 | 1.21 | 23205 |
| 7 | Arg | 0 | C_6_H_14_N_4_O_2_ | 174.1115 | 175.1187 | 0.6 | 21861 |
| 8 | DL-Arginine | 5 | C_6_H_14_N_4_O_2_ | 174.1115 | 175.1187 | 0.6 | 21861 |
| 9 | 6-Isoinosine | 5 | C_10_H_12_N_4_O_5_ | 268.0808 | 269.0881 | 1.73 | 20995 |
| 10 | Glutathione oxidized | 22 | C_20_H_32_N_6_O_12_S_2_ | 612.1527 | 613.16 | 1.04 | 19164 |
| 11 | D-Phenylalanine | 7 | C_9_H_11_NO_2_ | 165.0791 | 166.0864 | 2.46 | 18122 |
| 12 | Moracenin C | 0 | C_45_H_44_O_11_ | 760.2867 | 783.2759 | 25.95 | 16583 |
| 13 | Acankoreoside J | 9 | C_47_H_74_O_20_ | 958.4726 | 959.4798 | 8.34 | 15756 |
| 14 | 2-Aminoacetophenone | 3 | C_8_H_9_NO | 135.0687 | 136.076 | 1.21 | 14798 |
| 15 | Deltaline | 7 | C_27_H_41_NO_8_ | 507.2814 | 530.2707 | 4.49 | 13444 |
| 16 | Tubuloside D | 13 | C_43_H_54_O_23_ | 938.3021 | 939.3094 | 25.95 | 13401 |
| 17 | 4-Hydroxyquinoline | 2 | C_9_H_7_NO | 145.0527 | 146.06 | 3.63 | 12947 |
| 18 | Icariin | 3 | C_33_H_40_O_15_ | 676.2341 | 699.2233 | 24.69 | 12663 |
| 19 | D-pantothenic acid | 1 | C_9_H_17_NO_5_ | 219.1107 | 220.1179 | 2.99 | 12540 |
| 20 | DL-Tyrosine | 11 | C_9_H_11_NO_3_ | 181.0737 | 182.081 | 1.2 | 12512 |
| 21 | p-Tolualdehyde | 3 | C_7_H_6_O_2_ | 122.0371 | 123.0444 | 1.21 | 11414 |
| 22 | Riboflavin (Vitamin B2) | 10 | C_17_H_20_N_4_O_6_ | 376.1381 | 377.1453 | 5.19 | 10909 |
| 23 | Heterodendrin | 8 | C_11_H_19_NO_6_ | 261.121 | 262.1283 | 0.81 | 10506 |
| 24 | Moracenin C | 0 | C_45_H_44_O_11_ | 760.2864 | 783.2757 | 27.68 | 8415 |
| 25 | Stearidonic acid | 0 | C_18_H_28_O_2_ | 276.2089 | 277.2162 | 12.69 | 8315 |
| 26 | Coumarin | 4 | C_9_H_6_O_2_ | 146.037 | 147.0442 | 1.21 | 8156 |
| 27 | Moracenin C | 0 | C_45_H_44_O_11_ | 760.2867 | 783.2759 | 26.11 | 7936 |
| 28 | 23-epi-26-Deoxyactein | 5 | C_37_H_56_O_10_ | 660.3892 | 683.3784 | 6.54 | 7484 |
| 29 | (25R)-Ruscogenin-1-O-β-D-xylopyranosyl (1→3)-β-D-fucopyranoside | 1 | C_38_H_60_O_12_ | 708.4096 | 731.3989 | 5.61 | 7308 |
| 30 | Guanine | 3 | C_5_H_5_N_5_O | 151.0494 | 152.0567 | 1.72 | 7100 |
| 31 | Yadanzioside P | 1 | C_34_H_46_O_16_ | 710.279 | 733.2682 | 27.93 | 6997 |
| 32 | Icariin | 9 | C_33_H_40_O_15_ | 676.2354 | 699.2246 | 25.39 | 6977 |
| 33 | Fucoxanthin | 1 | C_42_H_58_O_6_ | 658.4223 | 681.4115 | 8.26 | 6584 |
| 34 | Ephedradine C | 13 | C_30_H_40_N_4_O_5_ | 536.3026 | 559.2918 | 4.74 | 6483 |
| 35 | Tamariscinoside B | 5 | C_26_H_36_O_12_ | 540.222 | 541.2293 | 26.83 | 6127 |
| 36 | Kadsurenin B | 0 | C_20_H_22_O_5_ | 342.1465 | 365.1357 | 17.29 | 5918 |
| 37 | Icariin | 0 | C_33_H_40_O_15_ | 676.2333 | 699.2225 | 24.1 | 5822 |
| 38 | Prosapogenin 4 | 9 | C_48_H_75_NO_17_ | 937.5021 | 960.4913 | 8.15 | 5785 |
| 39 | 11-O-p-Coumarylnepeticin | 0 | C_39_H_56_O_4_ | 588.4206 | 589.4278 | 21.34 | 5589 |
| 40 | Indole-3-carboxylic acid | 1 | C_9_H_7_NO_2_ | 161.0479 | 162.0551 | 3.36 | 5420 |
| 41 | Moracenin C | 0 | C_45_H_44_O_11_ | 760.2859 | 783.2751 | 24.7 | 5275 |
| 42 | Mulberrofuran Q | 2 | C_34_H_24_O_10_ | 592.1345 | 615.1237 | 24.85 | 5193 |
| 43 | Adenine | 1 | C_5_H_5_N_5_ | 135.0548 | 136.062 | 0.78 | 5113 |
| 44 | Noroxyhydrastinine | 4 | C_10_H_9_NO_3_ | 191.0582 | 192.0654 | 6.41 | 4935 |
| 45 | (-)-Olivil-4',4''-di-O-β-D-glucopyranoside | 0 | C_32_H_44_O_17_ | 700.2605 | 723.2497 | 25.96 | 4760 |
| 46 | Kadsurenin B | 0 | C_20_H_22_O_5_ | 342.1468 | 365.136 | 17.45 | 4624 |
| 47 | Delbruine | 0 | C_25_H_39_NO_7_ | 465.2711 | 466.2784 | 12.38 | 4537 |
| 48 | Noroxyhydrastinine | 4 | C_10_H_9_NO_3_ | 191.0585 | 192.0658 | 6.66 | 4250 |
| 49 | Quinatoside D | 19 | C_39_H_60_O_11_ | 704.4104 | 705.4176 | 7.84 | 4240 |
| 50 | Sanleng acid | 0 | C_18_H_34_O_5_ | 330.2404 | 353.2296 | 12.69 | 4185 |
